# Deciphering host lysosome-mediated elimination of *Plasmodium berghei* liver stage parasites

**DOI:** 10.1038/s41598-019-44449-z

**Published:** 2019-05-28

**Authors:** L. Niklaus, C. Agop-Nersesian, J. Schmuckli-Maurer, R. Wacker, V. Grünig, V. T. Heussler

**Affiliations:** 1 Institute of Cell Biology, University of Bern, Bern, Switzerland; 2 Graduate School of Cellular and Biomedical Sciences, University of Bern, Bern, Switzerland; 3 Department of Molecular and Cell Biology, Henry M. Goldman School of Dental Medicine, Boston University, Boston, MA USA

**Keywords:** Cell biology, Microbiology

## Abstract

Liver stage *Plasmodium* parasites reside in a parasitophorous vacuole (PV) that associates with lysosomes. It has previously been shown that these organelles can have beneficial as well as harmful effects on the parasite. Yet it is not clear how the association of lysosomes with the parasite is controlled and how interactions with these organelles lead to the antagonistic outcomes. In this study we used advanced imaging techniques to characterize lysosomal interactions with the PV. In host cells harboring successfully developing parasites we observed that these interaction events reach an equilibrium at the PV membrane (PVM). In a population of arrested parasites, this equilibrium appeared to shift towards a strongly increased lysosomal fusion with the PVM witnessed by strong PVM labeling with the lysosomal marker protein LAMP1. This was followed by acidification of the PV and elimination of the parasite. To systematically investigate elimination of arrested parasites, we generated transgenic parasites that express the photosensitizer KillerRed, which leads to parasite killing after activation. Our work provides insights in cellular details of intracellular killing and lysosomal elimination of *Plasmodium* parasites independent of cells of the immune system.

## Introduction

Malaria is a life-threatening disease, which is prevalent in more than 90 countries. The causative agents of malaria are unicellular eukaryotic organisms of the genus *Plasmodium*. *Plasmodium* parasites have caused more than 200 million cases and 435’000 deaths in the year 2017^1^. Transmission of the parasites occurs during the bite of an infected female *Anopheles* mosquito. The mosquito injects a small number of *Plasmodium* sporozoites into the skin of the mammalian host. Only one third of injected sporozoites successfully reach the liver to infect hepatocytes^2^. The liver stage is an essential stage, during which the parasites develop and produce merozoites. Once merozoites are released into the blood stream, they immediately infect red blood cells. Due to the low number of successfully infected hepatocytes (usually less than 100 per infectious mosquito bite), the liver stage is considered as a bottleneck in the life cycle of the parasite and as such is an important target for intervention^3,4^.

The clinically silent *Plasmodium* liver stage starts when the sporozoite invades a hepatocyte by invaginating the host cell plasma membrane, resulting in the formation of a parasitophorous vacuole (PV). The parasite resides and develops inside the PV throughout liver stage development. The PV membrane (PVM) is an important interface between the parasite and the host cell and becomes extensively modified by the parasite upon infection^5,6^. The modifications include a complete change of the protein composition of the PVM and the generation of an expansive and highly dynamic membranous tubovesicular network (TVN) protruding from the PVM into the host cell cytoplasm^7,8^. The extensive modifications of the PVM aid the parasite to import nutrients from the host cell and to export waste products. Moreover, recently it has been suggested that the TVN is used by the parasite to escape antimicrobial intracellular host cell responses, such as autophagy and autophagy-related pathways. Upon infection, the PVM of invading *Plasmodium* parasites becomes immediately decorated with host cell proteins of the autophagy pathway^3,8–11^. It has been shown that the parasite sheds the autophagy marker LC3 (microtubule-associated protein 1 light chain 3) from the PVM into the TVN and deposits it there^7^. Because the molecular details of this observed parasite labeling and the shedding phenomenon differ from canonical autophagy, we introduced the term *Plasmodium*-associated autophagy-related (PAAR) response^12^. Since only half of the liver stage parasites develop to mature merozoites, it has been suggested that the other half is eliminated by PAAR responses^3^. In contrast to PAAR response-mediated parasite elimination, activation of canonical autophagy in the host cell cytoplasm can support parasite development by providing additional nutrients^3,9,10^. When lysosomes fuse with autophagosomes, the content is degraded, and the released nutrients can support parasite growth. Because of this potentially deleterious and beneficial effects of PAAR and canonical autophagy, respectively, it is important for the parasite to control signaling pathways that result in autophagy and lysosome recruitment.

Lysosomes are organelles of the endocytic pathway that mature from endosomes, while endosomes are formed during endocytosis of extracellular material. Extracellular material reaches the lysosomes via endosomes and intracellular material via autophagosomes. In lysosomes, the cargo is degraded, and nutrients are released. To fulfill their function, lysosomes have an acidic luminal environment with activated hydrolases, and a typical membrane and protein composition. A typical lysosome marker protein is lysosome-associated membrane protein 1 (LAMP1), which is also a key marker protein for late endosomes (LE). Not surprisingly, vesicles of the endo-lysosomal pathway can be exploited by intracellular pathogens as a nutrient source. However, at the same time they have to avoid lysosomal degradation, as it is known for *Salmonella*, *Mycobacterium tuberculosis*, and *Legionella pneumophila*^13–15^. During *P. berghei* liver stage infections, it has been shown that LE and lysosomes localize around the parasite directly after sporozoite invasion^8,16^. It is not entirely clear if there is direct physical interaction of the PVM with lysosomes/LE, but the functionality of the late endocytic pathway in host cells is important for the extraordinary fast growth of the *Plasmodium* parasite^16,17^. One possible explanation is that the parasites exploit endocytic vesicles, including lysosomes, as a source of nutrients. This hypothesis is based on the observation that material of the host cell endocytic pathway appear to reach the PV and then the parasite^16^. Contrarily to the beneficial effects, interactions of LE and lysosomes with the PVM of *Plasmodium* liver stage parasites can lead to parasite killing^3,7,9,18^. This is supported by the fact that acidic vesicles are found around the parasite^16^ suggesting lysosomal attack by the host cell. Moreover, it has been suggested that parasites, which can control association with lysosomes are more likely to become mature schizonts and complete liver stage^3^. In successfully developing parasites, PVM components including autophagy marker proteins are transported to the TVN and finally dispatched in TVN-derived vesicles. It has been suggested that the shed vesicles are then targeted by lysosomes. Thus they keep the host cell degradative pathways away from the parasites and at the same time generate additional nutrients for parasite growth^7^.

In the present study, we further explored the fact that a proportion of parasites develop successfully whereas others are eliminated. We quantified LAMP1 association with the PVM and analyzed interaction of LE and lysosomes with the PVM by super-resolution microscopy. Additionally, we characterized the distribution of LE and lysosomes in a spatio-temporal manner with the help of the photo-convertible fluorescent protein mEos3.2 fused to LAMP1. We also analyzed parasite elimination in more detail and could show that a proportion of parasites are eliminated by enhanced fusion of the PVM with lysosomes and subsequent acidification. Furthermore, we generated an optogenetic approach to specifically kill liver stage parasites and then followed the interaction of the targeted parasites with lysosomes.

## Results

### Lysosomal marker protein LAMP1 colocalizes with the PVM and TVN of liver stage parasites

**Figure 1:**
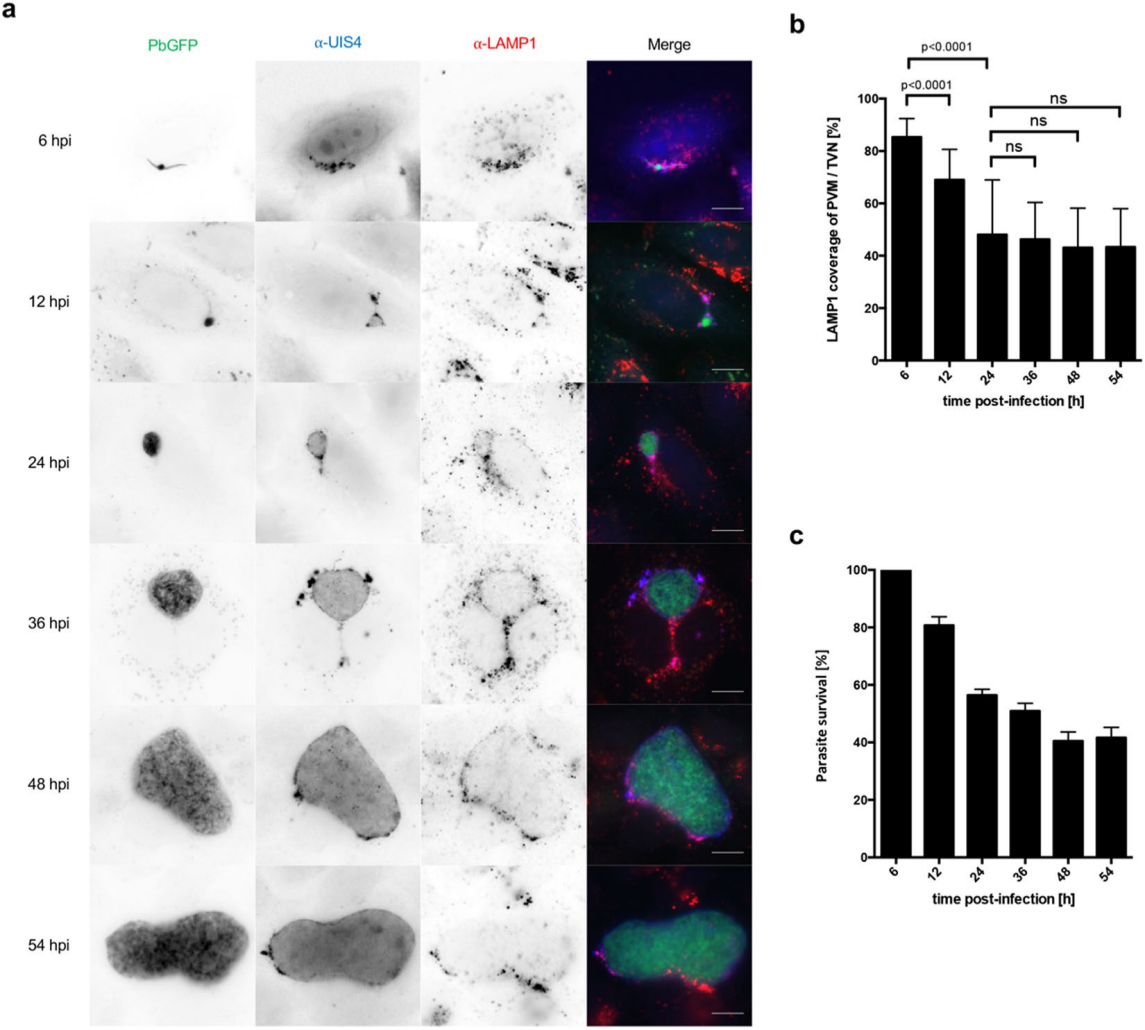
Lysosomal marker protein LAMP1 colocalizes with the PVM and TVN of liver stage parasites. Lysosomes at the PVM/TVN decrease during the development until the early schizont stage. (**a**) Widefield microscopy of HeLa cells infected with GFP-expressing *P. berghei* parasites (PbGFP, green). Cells were fixed and stained with α-UIS4 (blue; Alexa350) and α-LAMP1 (red; Alexa594). Scale bars are 10 µm. (**b**) Quantification of LAMP1 colocalization with the PVM/TVN. Column graph of the Mander’s colocalization coefficient of LAMP1 compared to UIS4. Mander’s values are between 0 and 1. High colocalization equals 1, while no colocalization equals 0. Colocalization of 1 means 100% coverage of UIS4 with LAMP1. The graph shows mean and standard deviation of two independent experiments. N ≥ 100 parasites. A student’s t test was used to determine p values. (**c**) Quantification of parasite survival. Column graph of relative number of parasites normalized to the 6 hpi time point. The graph shows mean and standard deviation of three independent experiments. N ≥ 460 parasites.

We and others have previously shown that lysosomes/LE are attracted to intracellular *P. berghei* liver stage parasites^3,9,17^ and that PVM material is shed into the TVN to keep detrimental material away from the vulnerable replicative center of the parasite^7^. We hence wanted to know whether there is direct interaction of lysosomes with the parasite or whether the parasite completely blocks such lysosome association events with the PVM/TVN. We therefore quantified the amount of lysosomes/LE associated with the PVM/TVN at different developmental stages of the *P. berghei* liver parasite. Using the UIS4 (up-regulated in infective sporozoites gene 4) protein as a PVM/TVN marker, LAMP1-positive lysosomes/LE were found associated with the PVM/TVN as soon as 6 hours post-infection (hpi) (Fig. 1a). We observed a persistent colocalization of the two signals across the whole liver stage development (Fig. 1a). However, the extent of the overlap seemed to change between the early time points compared to later ones. To quantify the degree of UIS4 and LAMP1 colocalization and thus the colocalization of lysosomes/LE with the PVM, we evaluated the Mander’s colocalization coefficient^19^ to calculate the percentage of UIS4 signal overlapping with LAMP1 signal. Quantification of UIS4 and LAMP1 overlap revealed that more than 85% of the PVM/TVN is covered with LAMP1 at 6 hpi (Fig. 1b). The amount of LAMP1 on the PVM/TVN decreases during the early liver stage until it reaches a constant proportion of approximately 40% (Fig. 1b). To analyze if the coverage of the PVM with LAMP1 correlates with parasite survival, we have analyzed the relative number of parasites from 6 hpi until the late liver stage at 54 hpi (Fig. 1c). The quantifications confirmed that the survival of parasites decreases between 6 hpi and 24 hpi to less than 60%, while the number of surviving parasites is relative stable during the late liver stage (Fig. 1c).

The here presented quantification of PVM coverage with the lysosomal marker protein LAMP1 suggested that a constant association of lysosomes with the PVM is tolerated by the parasite as the relative number of surviving parasites during the schizont stage (24 hpi onwards) is stable even in the presence of LAMP1. Lysosomal association might support parasite growth by releasing additional nutrients into the PV. Furthermore, we show that the decrease in parasite numbers is strongest during the early liver stage and thus correlates with the strongest LAMP1 association with the PVM/TVN.

### Super-resolution microscopy reveals that LAMP1-positive vesicles directly associate with the PVM in HeLa cells as well as in primary murine hepatocytes

**Figure 2:**
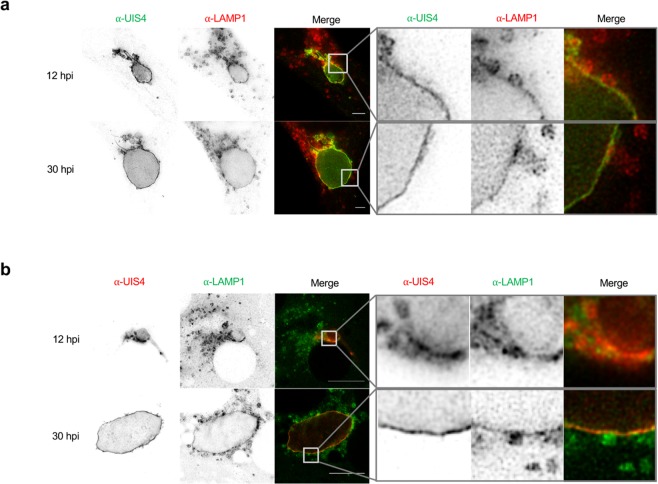
Super-resolution microscopy reveals that LAMP1-positive vesicles directly associate with the PVM in HeLa cells as well as in primary murine hepatocytes. STED images of HeLa cells (**a**) and primary murine hepatocytes (**b**) infected with mCherry-expressing *P. berghei* (PbmCherry). Images were taken using the Leica TCS SP8 STED microscope. Scale bars are 10 µm. (**a**) HeLa cells were fixed and stained with α-UIS4 (green; Oregon Green 488) and α-LAMP1 (red; Alexa532). (**b**) Primary murine hepatocytes were fixed and stained with α-UIS4 (red; Alexa532) and α-LAMP1 (green; Oregon Green 488). Enlargements of the indicated boxed part represented next to each image show possible fusion of vesicles with the PVM. Furthermore, it shows that LAMP1 localizes in the PVM of the parasite. Note that the resolution in the primary murine hepatocytes is decreased compared to the HeLa cells. The cultivation of primary murine hepatocytes requires coating with collagen, which partially interferes with the imaging. Additionally, primary murine hepatocytes are relatively thick compared to HeLa cells, which also decreases the resolution.

To investigate the interaction and possible fusion events of LAMP1-positive vesicles (LE and lysosomes) with the PVM/TVN we engaged super-resolution microscopy namely STimulated Emission Depletion (STED) microscopy^20^. STED imaging was performed on *P. berghei* infected HeLa cells stained with anti-UIS4 and anti-LAMP1 to visualize the PVM/TVN and lysosomes/LE, respectively (Fig. 2a). We observed a close association of LAMP1 with UIS4 at the trophozoite stage (12 hpi) and the schizont stage (30 hpi). The enlargements of the indicated boxed part in each image emphasize a partial colocalization of LAMP1-positive vesicles with the PVM (Fig. 2a; right panels). Further, we observed a concentration of LAMP1 signal in very close proximity of the vesicles to the PVM suggesting possible fusion events.

Since HeLa cells are not natural host cells for *Plasmodium* liver stage parasites, we investigated the association of lysosomes/LE with the PVM in *P. berghei* infected primary murine hepatocytes. STED imaging of these cells corroborated the data obtained in HeLa cells showing a close association of lysosomes/LE with the PVM (Fig. 2b). The enlargements of the indicated boxed part in the images show a magnification of LAMP1-positive lysosomes/LE next to the PVM of trophozoite stage parasites (12 hpi) and schizonts (30 hpi) (Fig. 2b, right panels).

The STED imaging of infected murine hepatocytes and HeLa cells strongly suggest that lysosomes/LE directly interact and possibly fuse with the PVM. It also provides further evidence that HeLa cells are reasonable model cells for *P. berghei* infection to study lysosome-PVM interactions.

### LAMP1 is shed from the PVM towards the TVN of the parasite

**Figure 3:**
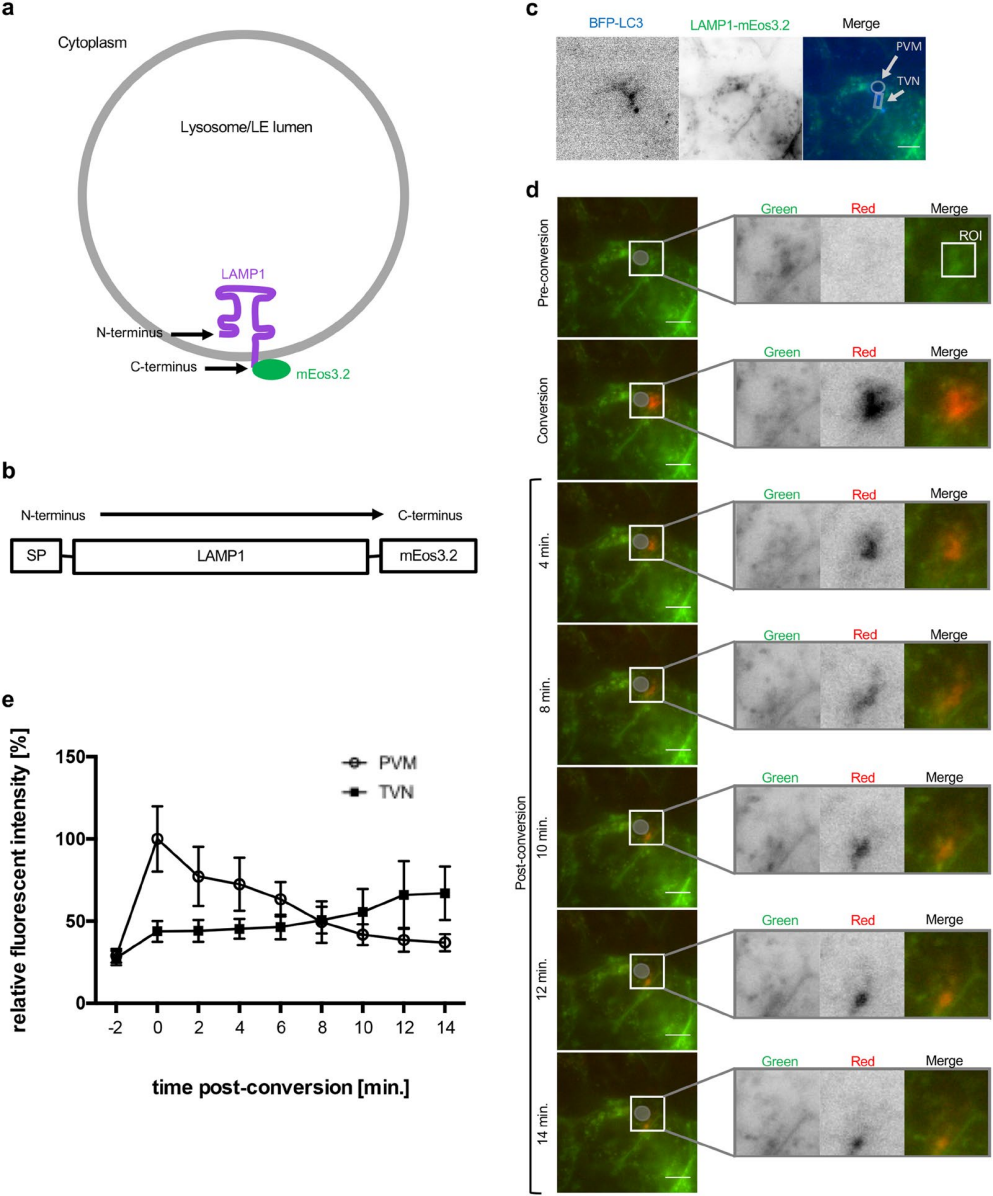
LAMP1 is shed from the PVM towards the TVN of the parasite. (**a**) A lysosome is represented with LAMP1 in its membrane. The N-terminus resides in the lysosomal lumen and the C-terminus points into the cytoplasm. Therefore, mEos3.2 faces the cytoplasm. (**b**) LAMP1 is represented from its N- to the C-terminus. The N-terminus contains a signal peptide (SP). mEos3.2 is fused to the C-terminus of LAMP1. (**c**,**d**) HeLa cells were transfected with BFP-LC3 plus LAMP1-mEos3.2. The transfected cells were infected with wild-type *P. berghei* (PbWT) and allowed to develop until 24 hpi before the imaging started. (**c**,**d**) Show different staining of exactly the same cell (**c**) BFP-LC3 was used to identify infected cells. The inverse images show BFP-LC3 (blue) and LAMP1-mEos3.2 (green). In the merged image, the parasite was identified by the BFP-LC3 in the parasite’s PVM. The circle depicts the PVM while the TVN is surrounded by a box. (**d**) LAMP1-mEos3.2, which is associated with the PVM was converted in the region of interest (ROI). The parasite is indicated by a grey circle. The ROI is only represented in the enlargement (merge). Enlargements of the indicated boxed part represented next to each image show that converted lysosomes (red) are moving from the PVM towards the TVN. The image shows one representative parasite out of 10 from three independent experiments. Scale bars are 10 µm. (**e**) Quantification of relative fluorescent intensity in the red channel at the PVM and TVN, respectively (N = 10; mean ± SD). The graph shows that the relative red fluorescence in the PVM decreases, while it increases in the TVN.

We have shown before that PVM incorporated autophagy marker proteins are shed into the TVN and it was hypothesized that this could potentially restrict lysosome-mediated elimination of the parasite^7^. To investigate this hypothesis in more depth, we first wondered whether the LAMP1-positive vesicles, which interact with the PVM are indeed acidic and therefore potentially harmful to the parasite. Experiments with a newly generated LAMP1 tandem pH-sensing construct (Figs S1 and S2) indicated that mature (acidic) lysosomes directly associate with the PVM of the parasites (Figs S3 and S4). In a next set of experiments, we investigated whether the parasite uses PVM shedding to control interaction with lysosomes. To analyze the dynamics of LAMP1 in *P. berghei* infected HeLa cells, we used the photo-convertible fluorescence protein mEos3.2. This protein initially is in its green fluorescent state. Upon irradiation with violet light of 405 nm, mEos3.2 irreversibly converts into its red state^21^. Tagging LAMP1 with mEos3.2 allows investigating the localization of LAMP1 in a spatio-temporal manner. LAMP1 is a transmembrane protein with its C-terminus facing the host cell cytoplasm and the N-terminus in the LE/lysosomal lumen (Fig. 3a). To prevent mEos3.2 from being exposed to the acidic environment of the lysosomal lumen it was fused to the C-terminus of LAMP1 (Fig. 3a,b).

First, we confirmed correct lysosomal localization of the newly generated LAMP1-mEos3.2 in non-infected HeLa cells (Fig. S1f). We then studied LAMP1 dynamics in infected cells and questioned whether LAMP1-positive PVM material is shed into the TVN, like it was shown earlier for the autophagy marker protein LC3^7^. To identify infected cells prior to photo-conversion, HeLa cells were co-transfected with BFP-LC3 in addition to LAMP1-mEos3.2 (Fig. 3c), because LC3 strongly labels the PVM of *P. berghei* parasites^3,8,10^. After photo-conversion, LAMP1-mEos3.2 was in its red state in the region of interest (ROI) and could be traced (Fig. 3d). During the observation time of 14 minutes, photo-converted LAMP1-mEos3.2 moved from the PVM towards the TVN (Fig. 3d). To quantitatively analyze the movement of LAMP1-mEos3.2 from the PVM into the TVN, we measured the mean intensity of the converted red fluorescence at the PVM or TVN respectively during the observation time (Fig. 3e). This revealed that the fluorescence intensity decreased over time in the PVM, while it increased in the TVN and thus shows similar dynamics as PVM-associated LC3^7^.

These experiments strongly suggest that LAMP1-positive material is shed from the PVM into the TVN. Together with the observation that the PVM coverage of LAMP1 reaches a stable level (Fig. 1b), it likely represents an equilibrium, where new lysosomes/LE constantly associate with the PVM, while others are shed into the TVN probably protecting the parasite from an overwhelming lysosomal attack.

### LAMP1 is trapped in the TVN of the parasite

**Figure 4:**
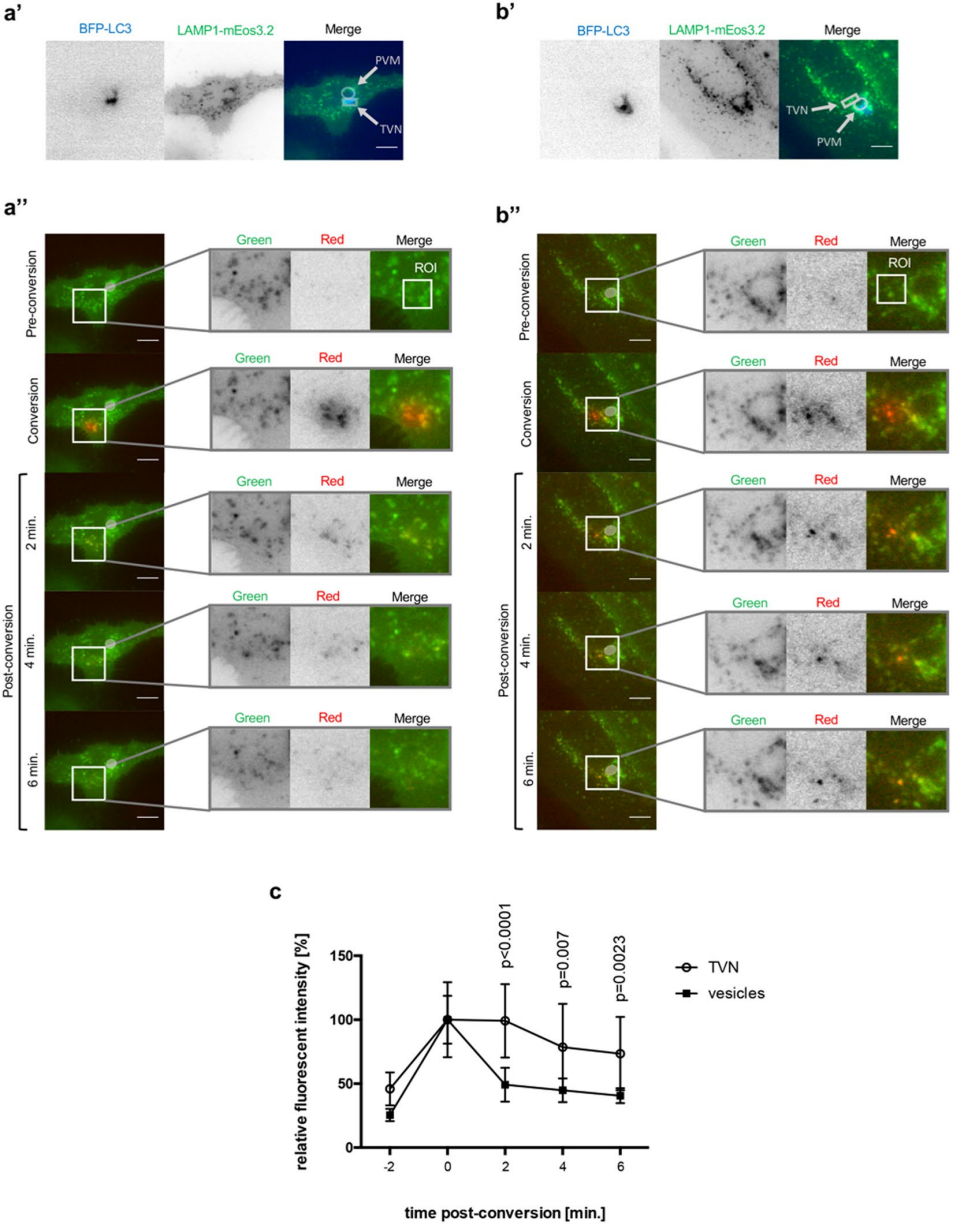
LAMP1 is trapped in the TVN of the parasite. HeLa cells were transfected with BFP-LC3 plus LAMP1-mEos3.2. Transfected cells were infected with wild-type *P. berghei* (PbWT) and imaged at 24 hpi. (**a’**,**b’**) BFP-LC3 was used to identify infected cells. Inverse images show BFP-LC3 (blue) and LAMP1-mEos3.2 (green). In the merged images one can recognize, where the parasite resides in the host cell due to BFP-LC3 in the parasite’s PVM. Circle depicts the PVM while the TVN is surrounded by a square box. (**a”**,**b”**) Parasites are indicated by a bright grey circle. The ROI is shown in the enlargement (merge). (**a’**,**a”**) Show the same infected cell. In a” LAMP1-mEos3.2 was converted outside of the TVN (enlargement, merge). (**b’**,**b”**) Show the same infected cell. In b” LAMP1-Eos3.2 was photo-converted in the TVN of the parasite as indicated with the ROI (enlargement, merge). One representative infected cell out of 10 from three independent experiments has been shown for each set of images. Scale bars are 10 µm. (**c**) Quantification of relative fluorescent intensity in the red channel (N = 10; mean ± SD). The p values were determined with a student’s t test.

To further analyze the shedding process, we next wanted to know whether LAMP1 is trapped in the TVN or moves freely between PVM and TVN. Consequently, we investigated the fate of LAMP1 at the TVN. Infected cells were again identified with the help of BFP-LC3 (Fig. 4a’,b’). As a control, we first analyzed LAMP1-positive vesicles in the cytoplasm of infected cells that are not associated with the PVM or the TVN (Fig. 4a”). Such non-parasite-associated LAMP1-mEos3.2-positive vesicles were photo-converted, and their movement was monitored. Short-time frame imaging revealed that the vesicles were distributed throughout the cell in less than 6 min. (Fig. 4a”). Next, we photo-converted LAMP1-mEos3.2 localizing to the TVN of the parasite. Tracing of the photo-converted LAMP1-mEos3.2 reveals that it is still detected in the TVN 6 min. post-conversion (Fig. 4b”). Quantification of these experiments confirmed that the red fluorescent signal of LAMP1-mEos3.2 quickly decreased if freely moving vesicles in the cytoplasm were analyzed, while it could be detected at a relatively constant rate in the TVN (Fig. 4c). Altogether, we show that random LAMP1-mEos3.2 positive vesicles move freely in the host cell’s cytoplasm, whereas LAMP1-mEos3.2 residing in the TVN of parasites is more static. These observations are supported by experiments, which show that LAMP1 accumulates at the TVN throughout the entire liver stage (Fig. S5).

### Parasite acidification and death correlates with strong rim-like LAMP1 signal around the parasite

**Figure 5:**
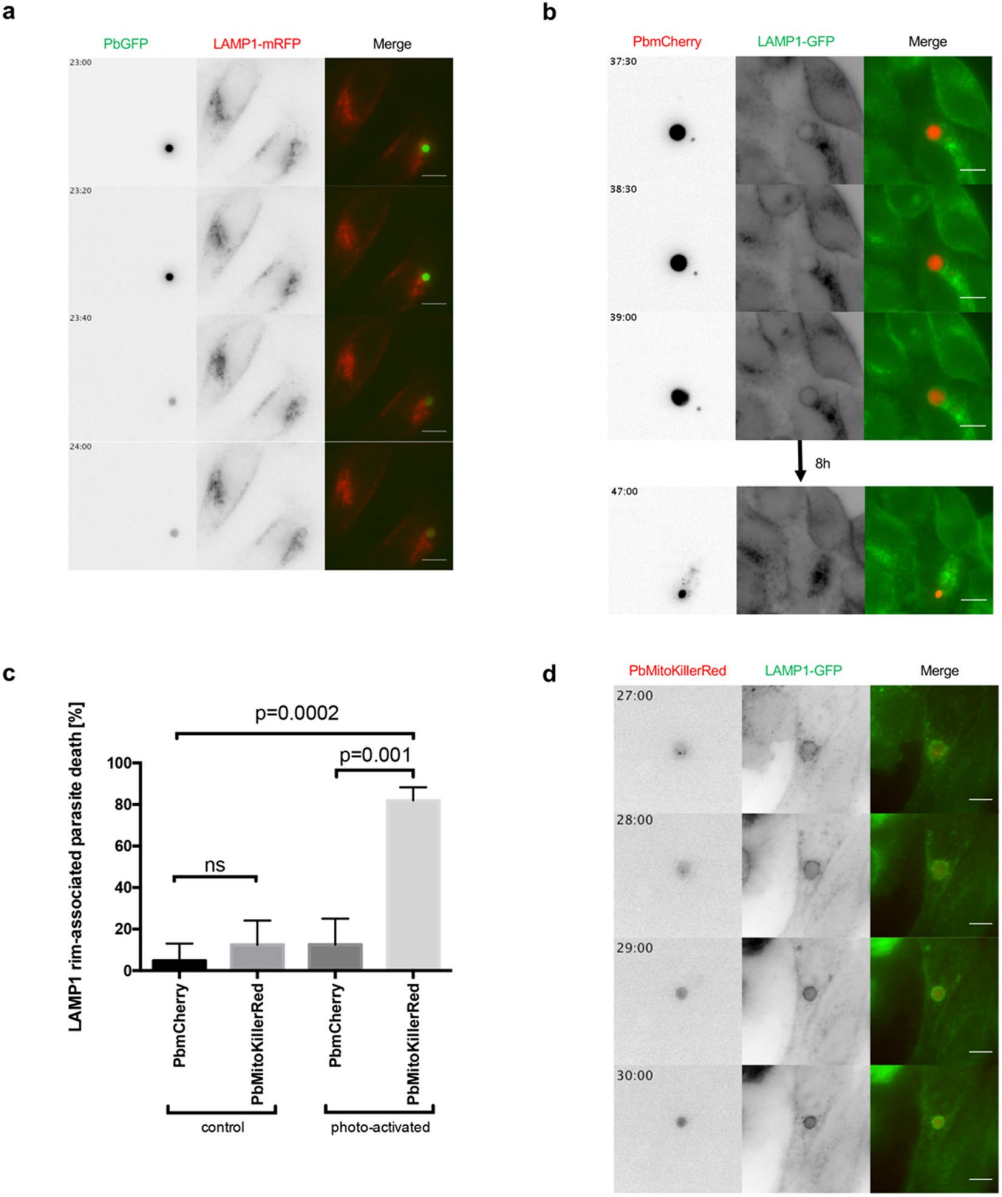
Parasite acidification and death correlates with strong rim-like LAMP1 signal around the parasite. (**a**) HeLa cells were transfected with LAMP1-mRFP (red) and infected with GFP-expressing parasites (PbGFP, green). As soon as LAMP1-mRFP forms a strong signal tightly around the parasite, the acid sensitive GFP signal disappears. (**b**) HeLa cells stably expressing LAMP1-GFP (green) were infected with mCherry-expressing parasites (PbmCherry, red). The formation of the strong LAMP1-GFP signal around the parasite takes less than 30 min. The representative parasite is smaller and degraded 8 h later. (**c**) Quantification of killed PbMitoKillerRed parasites compared to PbmCherry parasites without or with photo-activation. HeLa cells which stably express LAMP1-GFP were either infected with PbmCherry or PbMitoKillerRed. At 24 hpi the parasites where either not illuminated with green light (control) or illuminated with laser pulses of green light (photo-activation). Column graph of relative number of parasites eliminated after showing the LAMP1 rim pattern during the imaging (N ≥ 10; three independent experiments; mean ± SD). The p values were determined with a student’s t test. (**d**) HeLa cells stably expressing LAMP1-GFP (green) were infected with PbMitoKillerRed (red). LAMP1-GFP signal intensified tightly around the parasite after killing the parasite. Scale bars are 10 µm. Time stamps are in hh:mm post-infection.

After investigating the PVM shedding of potentially detrimental material by successfully developing parasites, we were next interested in parasites that arrest and are rapidly eliminated. To analyze whether arrested parasites are removed by lysosomal acidification, we took advantage of the pH sensitivity of GFP^22,23^ (Fig. S6). We infected HeLa cells expressing LAMP1-mRFP with parasites that constitutively express GFP in their cytoplasm (PbGFP). Subsequently, we analyzed parasites which showed a GFP signal at the beginning, but then had a decrease in detectable GFP signal over time, as this indicates acidification of the parasites’ cytoplasm and elimination (Figs 5a and S6). Such parasites are in contrast to normally developing parasites represented in Fig. 1a, which show a GFP signal throughout their entire development. Interestingly, acidified parasites exhibit a distribution of LAMP1 that differs markedly compared to the LAMP1 distribution in the PVM of successfully developing parasites (Movie 1a and Fig. 5a). Before the parasite loses GFP signal, LAMP1-mRFP can be observed to localize in vesicles closely around the parasite. Remarkably, just as the GFP signal fades, it is observed that LAMP1-mRFP localizes in a strong, smooth rim around the parasite, most likely the PVM. As the GFP signal disappears, the parasite cytoplasm becomes invisible and it is no longer possible to trace it. To follow the fate of parasites with a strong smooth LAMP1 rim around them, we used mCherry-expressing parasites (PbmCherry), as mCherry is stable in acidic conditions^24^ (Fig. 5b). HeLa cells stably expressing LAMP1 tagged with GFP at the C-terminus were infected with PbmCherry parasites. Long-term live imaging revealed that the change in the pattern of LAMP1 to the strong smooth LAMP1 rim happened within less than 30 min. We observed that these parasites are then indeed rapidly shrinking and are finally eliminated (Movie 1b and Fig. 5b).

To get a more detailed knowledge about elimination of *P. berghei* liver stage parasites, we generated a new parasite line that allows parasite killing in an optogenetic manner. These transgenic parasites express the photosensitizer protein KillerRed, which generates toxic reactive oxygen species (ROS) upon illumination with green light. Eukaryotic cells are most efficiently killed if KillerRed is expressed in their mitochondria or plasma membrane^25^. To generate parasites that mitochondrially express KillerRed (PbMitoKillerRed), we fused KillerRed with the mitochondrial targeting sequence of *P. berghei* citrate synthase^26^. After verifying integration of the plasmid into the *P. berghei* parasites’ genome and confirming mitochondrial expression (Fig. S7), we infected HeLa cells stably expressing LAMP1-GFP with the PbMitoKillerRed parasites. With these settings, it was possible to explore two main questions: Does parasite death correlate with the strong smooth LAMP1 rim around the parasite and does this result in parasite elimination? Quantifications revealed that PbMitoKillerRed parasites were indeed efficiently killed after illumination with green light (Fig. 5c). During the time imaged, 2% of PbmCherry parasites spontaneously exhibited a strong smooth LAMP1 rim, which was associated with parasite elimination, whereas 90% of PbMitoKillerRed parasites showed this phenotype upon illumination (Fig. 5c). Movie 1c and Fig. 5d show that, after illumination, LAMP1-GFP intensifies in a rim-like LAMP1 signal around the PbMitoKillerRed parasite followed by its elimination. Importantly, the appearance of the rim-like LAMP1 signal depends on PV acidification because it can be blocked by chloroquine treatment, which inhibits lysosomes and thus also PV acidification (Fig. S8). The observed acid-dependent appearance of the LAMP1 rim around killed PbMitoKillerRed parasite was similar to what has been observed in the relatively rare spontaneous acidification events (Fig. 5a). Importantly, PbmCherry parasites with the same rim-like LAMP1 signal were observed to be eliminated (Fig. 5b).

Conclusively, our data show that a population of arrested parasites exhibits a completely different LAMP1 localization pattern in the PVM compared to successfully developing parasites. While the here presented arrested parasites show a strong smooth LAMP1 rim in the PVM, successfully developing parasites most probably engage PVM shedding to restrict PVM labeling with potentially harmful material such as LAMP1 and associated lysosomes. The newly generated PbMitoKillerRed parasites will now allow further dissection of possible pathways and involved host cell factors.

## Discussion

Here we show that the localization of lysosomes differs substantially between a population of arrested liver stage *P. berghei* parasites and successfully developing parasites. Lysosomes appear to associate with all parasites, but the result of this association can have a totally different outcome. While a population of arrested parasites becomes acidified and is rapidly eliminated, successfully developing parasites do not show any acidification. Interestingly, we have shown here that the association of lysosome/LE with the PVM reaches an equilibrium suggesting that possible lysosome-PV interactions themselves are not harmful for the parasite. The fact that this equilibrium lasts until the late liver stage even indicates that the parasite might benefit from the interaction events with lysosomes. It is likely that the equilibrium is sustained by a balance between lysosomes/LE newly associating with the PVM and lysosomes/LE being shed first into the TVN and finally into the host cell cytoplasm. By maintaining an equilibrium, *P. berghei* liver stage parasites might avoid an excess of detrimental acidic and hydrolytic lysosomal content but at the same time profit from the lysosomal nutrients released into the PV. *P. berghei* is not the only protozoan parasite, which uses nutrients from lysosomes and at the same time avoids lysosomal elimination. *Leishmania* parasites even reside in lysosomes of macrophages and take up free sugars, lipids, peptides and amino acids from the lysosomal lumen via transporters in their plasma membrane^27,28^.

Apart from being nutritious, lysosomes and more specifically their membranes are a signal platform involved in pro-survival and autophagic signaling pathways. We have previously shown that the autophagy marker protein p62 (nucleoporin 62) is found on the PVM^3,11^. P62 is known to be able to activate the pro-survival factor mTORC1 (mammalian target of rapamycin complex 1) on the lysosomal membrane^29,30^. The complex mTORC1 is the gatekeeper of canonical starvation-induced autophagy^31,32^ and starvation-induced autophagy has been shown to have a strong positive impact on parasite liver load^3,9^. Furthermore, mTORC1 is part of the lysosomal nutrient sensing (LYNUS) machinery on the surface of lysosomes which signals the nutrient status to the mammalian nucleus^33^. Parasites might be in a better position to control these pathways, if lysosomes are in close proximity to them. Altogether, the balanced equilibrium of lysosomes at the PVM might allow the parasite to get the best out of this noxious yet nutritious organelle and allows it at the same time to control signaling pathways on the lysosomal surface to guarantee host cell survival.

The suggestion that the equilibrium is sustained by lysosomes/LE constantly associating with the PVM is supported by electron microscopy studies that showed vesicles of the endocytic pathway in close proximity to the parasites’ PVM^16^. Our super-resolution microscopy data extend these earlier findings in providing evidence that vesicles in close proximity to the PVM are LAMP1-positive and are therefore lysosomes/LE. Although LAMP1 localization in the PVM strongly suggests fusion with lysosomes, we realized that LAMP1 in the PVM often localized in a higher concentration close to the site where the lysosome/LE met the PV and does not seem to diffuse quickly in the PVM. A possibility is therefore a kiss-and-run fusion of lysosomes with the PV. Kiss-and-run fusion events do not lead to the complete fusion of membranes^34^, but still allow the transfer of some membrane material and lysosome content including lipids and other nutrients. After this event, the lysosomes separate again completely from the target membrane. A kiss-and-run fusion would allow the parasite to get access to some nutrients in the lysosomes but protects it from exposure to the full potentially harmful content.

**Figure 6:**
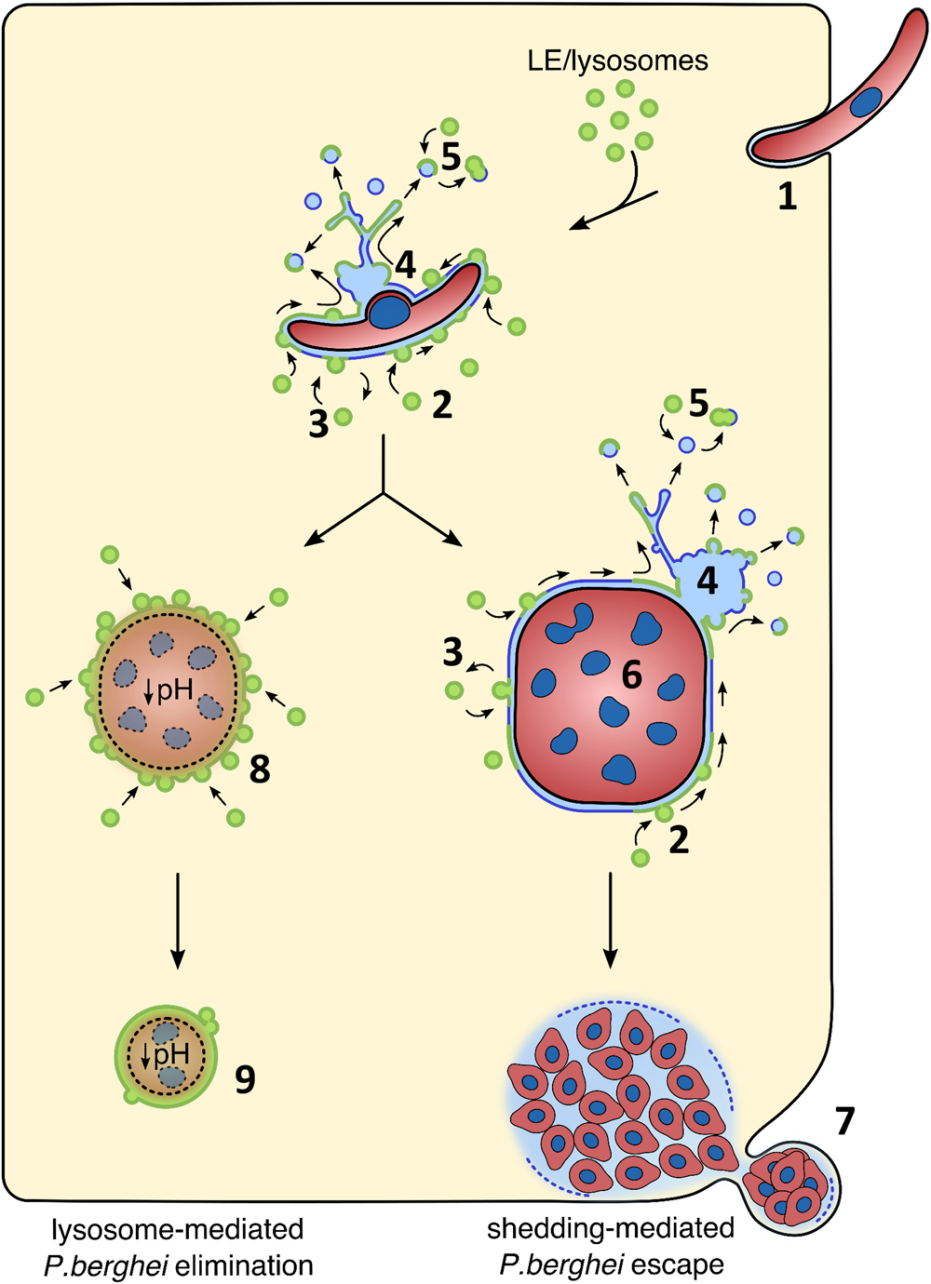
Graphical summary of the proposed lysosome dynamics at the PVM. Invading parasites (1) attract lysosomes/late endosomes to their PVM. Lysosome fusion with the PVM could either be complete (2) or follow a kiss-and-run strategy (3). Viable parasites are able to control fusion of lysosomes with the PVM by constant shedding of PVM material into the TVN (4) and TVN-derived vesicles (shedding-mediated escape) that can fuse with lysosomes to degrade the content that can then potentially be used by the parasite as nutrients (5). Successfully developing parasites (6) continue PVM shedding and reach an equilibrium of fusion and shedding until merozoites are formed that are packed in merosomes (7) and transported to the blood stream. Arrested parasites are not able to control lysosome fusion with the PVM (8) and thus the PV and finally the parasite become acidified and eliminated (lysosome-mediated elimination) (9). Green membrane = LAMP1 positive, blue membrane = original PVM/TVN membrane.

The constant association of lysosomes is very likely balanced by a constant shedding of PVM material towards the TVN. For the first time we provide evidence that this shedding includes also LAMP1 and therefore lysosome membrane material, indicating that PVM shedding is a defense mechanism to protect the parasite from an overwhelming attack of lysosomes. This has already been suggested earlier, but in that study the autophagy marker protein LC3 was investigated^7^. Here we provide direct evidence that LAMP1-positive vesicles become trapped in the TVN away from the parasite where acidification does potentially not harm the parasite. Similar to our observation, Grützke and colleagues reported an association of late endocytic compartments with the TVN^8^. There might be an additional advantage for the parasite in directing lysosomes towards TVN vesicles: acidification of such TVN-derived vesicles could help to digest the TVN’s cargo and therefore lead to an additional supply of nutrients for the intracellular parasite (Fig. 6). In agreement with this it has been shown that liver schizonts are smaller if lysosomal acidification is disrupted with Concanamycin A or chloroquine^3,16^. Our data suggest that the PV of successfully developing parasites is not acidified confirming earlier observations^16^ and supporting the shedding phenomenon as a defense mechanism against lysosomal elimination. However, the non-acidic nature of the PV has previously been discussed in another context. Real and colleagues have shown that the PVM resident protein UIS3 (up-regulated in infective sporozoites gene 3) binds into the hydrophobic pockets of the autophagy marker protein LC3 via a non-canonical LIR (LC3-interacting region) motif. It was proposed that the binding of UIS3 to LC3 subsequently inhibits the interaction of lysosomes with LC3-containing vacuoles like e.g. the PV^35^. However, Schmuckli-Maurer and colleagues have shown that LIR-containing proteins like p62 are found at the PVM of *P. berghei* liver stage parasites in an LC3-dependent manner^11^ and therefore the interaction capacity of PVM-resident LC3 is not completely abolished by binding to UIS3. Furthermore, it has been shown that lysosomes do associate with the PVM of *P. berghei* parasites even in ATG5-deficient cells, where LC3 is not found in the PVM^9,17^. Hence, the binding of UIS3 to LC3 does not completely explain the non-acidic nature of the PV in contrast to PVM shedding that potentially supports and explains the relative neutral pH in the PV of *P. berghei* liver stage parasites. The parasite possibly depends on both strategies in a complementary manner to efficiently evade lysosomal acidification. An additional explanation comes from the research of Bano and colleagues. They showed that the PVM of *P. berghei* parasites allows the free movement of substances with a size bellow 855 Da across the PVM through non-selective channels^5^. Protons, although charged molecules, with a size of 1 Da should therefore be able to freely cross the membrane and thus a pH gradient cannot be established. Nevertheless, lysosomal hydrolytic enzymes would not be able to freely pass the PVM and hence could damage the parasite. This might be a reason why developing parasites mediate lysosomal material transport towards the TVN and deposit it there for further degradation.

In contrast to the successfully developing parasites, we show that a population of arrested parasites are acidified and eliminated via lysosomal pathways. Remarkably, the LAMP1 localization of these arrested parasites looks considerably different compared to successfully developing parasites. The pattern of the PVM-associated LAMP1 in arrested parasites resembles a smooth rim wrapped tightly around the parasite. Furthermore, the TVN signal is no longer present. The reason might be that arrested parasites are most probably no longer able to maintain a functional PVM. Consequently, the equilibrium of lysosomes at the PVM might get out of balance: due to the continuous association of new lysosomes with the PVM and the loss of the TVN, these organelles would not be shed efficiently anymore and would accumulate at the PVM. With the help of the PbMitoKillerRed parasite line, we could confirm that the appearance of a strong, LAMP1-positive, clearly defined PVM-like signal depends on the health status of the parasite. When PbMitoKillerRed parasites are killed by exposing them to green light, they most likely stop exporting proteins into the PVM. Thus, the entire balanced system might collapse including the TVN as there is no more PVM shedding. In this case it is likely that lysosomes continually associate and fuse with the PV, which finally results in LAMP1 accumulation in the PVM and the observed phenomenon of a rim-like LAMP1-positive PVM. The importance of shedding, respectively the absence of it, is possibly represented in a situation when sporozoites enter host cells via a transient vacuole. Shedding and the establishment of a TVN can most likely not be performed in a transient vacuole. Therefore, such parasites are eliminated by lysosomal fusion if they cannot escape the transient vacuole^36^. The potential of lysosomes to fuse with the PVM of *Plasmodium* liver stage parasites has been suggested previously^18^. In that report, it was shown that, upon IFN-γ stimulation, lysosomes fuse with the PVM of *P. vivax* parasites through an autophagy-related pathway. This subsequently leads to parasite killing. Apart from not being able to maintain PVM shedding and the architecture of the TVN, there could theoretically be a further consequence of not being able to export proteins into the PVM. This is that arrested parasites are probably unable to maintain the porosity of the PVM through pore-forming proteins, which would subsequently no longer allow protons to freely pass across the PVM. In such a case, it is possible to establish a pH gradient in the PV and thus the arrested parasite became acidified. This is supported by the observation that blocking the export of proteins into the PVM, is lethal for the parasite^37^.

There is a very interesting scenario appearing from the recent literature and our work presented here, which can explain the observed phenomenon: liver stage parasites might maintain a very delicate balance of lysosome association with the PVM. As long as the parasite is able to actively export proteins into the PVM, the equilibrium is maintained. Parasites that become arrested in their development and that are metabolically inactive, are more likely eliminated by lysosomal attack. This might also explain how irradiated sporozoites, or genetically attenuated parasites are eliminated during the liver stage. If we understand the molecular machinery of lysosome association with the PVM better, we might be able to support the host cell defense machinery including the endo-lysosomal network to eliminate all parasites. Furthermore, it could not only be interesting to support the host cell intrinsic defense machinery, but also to use lysosomes/LE to deliver drugs via the endo-lysosomal pathway to the parasite. Finally, it is important to investigate on the machinery of lysosome-PV fusion events. Any vesicle fusion has to be orchestrated by a cascade of signaling events. Proteins, lipids and small molecules can mediate such signaling events. One potential mediator could be UIS4 as the recruitment of lysosomes/LE is restricted in UIS4-negative parasites^17^. It would now be interesting to investigate which additional proteins, lipids and small molecules are potentially involved in the interaction and fusion of lysosomes with the PV of parasites that display LAMP1 rim-associated death. The newly generated PbMitoKillerRed parasite line is a very useful tool to further analyze these molecular details of parasite elimination.

## Experimental Procedures

### Animal work statement

Balb/c mice used in the experiments were bred in the central animal facility of the University of Bern and were between 6 and 10 weeks of age. Experiments performed on laboratory animals were with strict accordance to the guidelines of the Swiss Tierschutzgesetz (TSchG; Animal Rights Laws) and approved by the ethical committee of the University of Bern. For mosquito blood feeds, mice were anesthetized with a mixture of ketasol and xylasol. For this purpose, 8.6 ml ketasol (100 mg/ml) were mixed with 5.8 ml xylasol (20 mg/ml) to a total volume of 30 ml in isotonic natrium chloride solution. The mice were anesthetized by intraperitoneally injecting 100 μl per 20 g body weight.

### Culture, treatment and *in vitro* infection of HeLa cells and primary hepatocytes

HeLa cells (European Cell Culture Collection) were cultured in minimum essential medium with Earle’s salts (MEM EBS; BioConcept, 1‐31F01‐I), supplemented with 10% FCS (GE Healthcare), 100 U penicillin (BioConcept), 100 μg/ml streptomycin (BioConcept), and 2 mM L-glutamine (BioConcept). Cells were cultured at 37 °C and 5% CO_2_ and split using Accutase (Innovative Cell Technologies) diluted 1:2 in phosphate-buffered saline (PBS; 137 mM NaCl, 2.7 mM KCl, 10 mM Na_2_HPO_4_, 1.8 mM KH_2_PO_4_, pH 7.4). Chloroquine treatment was performed in MEM complete supplemented with 10 μM chloroquine (Sigma-Aldrich C6628).

Primary murine hepatocytes were isolated and cultured as described elsewhere^3^. For infection of cells, salivary glands of infected *Anopheles stephensi* mosquitoes from 16–26 days after blood feeds were isolated and homogenized to release sporozoites. Sporozoites were incubated with cells in medium containing 25 μg/ml Amphotericin B (Amresco E437) for 2 h. Subsequently, cells were rinsed and incubated in MEM EBS complete containing 2.5 μg/ml Amphotericin B.

### Plasmids

LAMP1-RFP was a gift from Walther Mothes^38^ (Addgene plasmid #1817). To generate the pLAMP1-mEos3.2, the open reading frame (ORF) of mEos3.2 was amplified by PCR using the forward primer called mEos3.2-fw 5′-GGATCCAATGAGTGCGATTAAGCC-3′ and the reverse primer called mEos3.2-rev 5′-TCTAGATTATCGTCTGGCATTGTCAGG-3′ (Table 1) with the plasmid mEos3.2-ER-5 as template. The plasmid mEos3.2-ER-5 was a gift by Michael Davidson (Addgene plasmid #57455). After subcloning the cDNA via blunt ends into pJET1.2 (#K1232, Thermo Fisher Scientific, Reinach, Switzerland), the sequence was verified by the sequencing service of Microsynth AG in Balgach, Switzerland. Subsequently, mEos3.2 was cloned into the final vector pLAMP1-GFP after excising the GFP cDNA insert with the restriction enzymes BamHI and XbaI. The plasmid pLAMP1-GFP was kindly provided by John Brumell (Hospital for Sick Children, Canada).

**Table 1:** Primer list.

Name	Sequence (5′ ->3′)	Restriction site
c-ssu-fw	GTGTAGTAACATCAGTTATTGTGTG	
d-ssu-fw	ATACTGTATAACAGGTAAGCTGTTATTGTG	
pL0017-rev	TTTCCCAGTCACGACGTTG	
cd-ssu-rev	CTTAGTGTTTTGTATTAATGTCGATTTG	
mEos3.2-fw	GGATCCAATGAGTGCGATTAAGCC	BamHI
mEos3.2-rev	TCTAGATTATCGTCTGGCATTGTCAGG	XbaI
mClover3-fw	CGTCAGCAACCGGTATGGTGAGCAAGGGCGAGGAGCTGTTC	AgeI
mRuby3-rev	GAATTCCTTGTACAGCTCGTCCATG	EcoRI
SPofLAMP1-fw	CTCGAGATGGCGCCCCGCAGC	XhoI
SPofLAMP1-rev	ACCGGTTGCTGACGCACAATGCAT	AgeI
LAMP1ΔSP-fw	TGTACAAGGAATTCGCAATGTTTATG	EcoRI
LAMP1-rev	TCTAGATTAGATAGTCTGGTAGCCTGCG	XbaI
KillerRed-fw	TGGATCCATGGGTTCAGAG	BamHI
KillerRed-rev	TTCTAGATTAATCCTCGTCGCTACC	XbaI
LAMP1-GFP-fw	ATCGGACCGATGGCGCCCCGCAG	RsrII
LAMP1-GFP-rev	ATCGGTCCGTTACTTGTACAGCTCG	RsrII
mTurquoise2-fw	AT(GGATCC)ACCGGTCGCCACCATGGTGAGCAAGGGCGAG	BamHI
mTurquoise2-rev	AT(TCTAGA)TTACTTGTACAGCTCGTCCATG	XbaI

To generate pEBFP2-LC3, the cDNA of human LC3B was excised from pEGFP-hLC3B with EcoRV and KpnI. The plasmid pEGFP-hLC3B was a kind gift from Johji Inazawa^39^. The excised cDNA of LC3B was ligated into pEBFP2-C-puro which was linearized with the same restriction enzymes.

To generate pmClover3-mRuby3-LAMP1, the cDNA of mClover3-mRuby3 was amplified via PCR from the template pKanCMV-mClover3-mRuby3, which was a gift of Michael Lin^40^ (Addgene plasmid #74252). To amplify the cDNA of mClover3-mRuby3, we used the forward primer mClover3-fw 5′-CGTCAGCAACCGGTATGGTGAGCAAGGGCGAGGAGCTGTTC-3′ and the reverse primer mRuby3-rev 5′-GAATTCCTTGTACAGCTCGTCCATG-3′ (Table 1). Furthermore, we amplified the sequence of the signal peptide (SP) of LAMP1 by PCR. To do so, we used the forward primer 5′-CTCGAGATGGCGCCCCGCAGC-3′, the reverse primer SPofLAMP1-rev 5′-ACCGGTTGCTGACGCACAATGCAT-3′ and pLAMP1-GFP as the template (Table 1). The sequence of the SP and the cDNA of mClover3-mRuby3 were then fused by fusion PCR with the help of overlapping sequences. This resulted in the PCR product SP-mClover3-mRuby3. We then further amplified the cDNA of LAMP1 without the SP (LAMP1 Δ SP) using pLAMP1-GFP as the template, the forward primer LAMP1ΔSP-fw 5′-TGTACAAGGAATTCGCAATGTTTATG-3′ and the reverse primer LAMP1-rev 5′-TCTAGATTAGATAGTCTGGTAGCCTGCG-3′ (Table 1). The PCR products SP-mClover3-mRuby3 and LAMP1 Δ SP were then used for a further fusion PCR, which led to the fused sequence of SP-mClover3-mRuby3-LAMP1. The sequence SP-mClover3-mRuby3-LAMP1 was subcloned via blunt ends into pJET1.2, the sequence was verified by the sequencing service of Microsynth AG in Balgach, Switzerland. SP-mClover3-mRuby3-LAMP1 was then cloned into pLAMP1-GFP after excising LAMP1-GFP with the restriction enzymes XhoI and XbaI.

To generate pLAMP1-mTurquoise2, we amplified the cDNA of mTurquoise2 with the forward primer mTurquoise2-fw 5′-ATGGATCCACCGGTCGCCACCATGGTGAGCAAGGGCGAG-3′ and the reverse primer mTurquoise2-rev 5′-ATTCTAGATTACTTGTACAGCTCGTCCATG-3′. After subcloning the cDNA into pJET1.2 and verifying the sequence, we excised the cDNA of mTurquoise2 with BamHI and XbaI. The DNA fragment was then ligated into the final vector pLAMP1-GFP after excising GFP with the restriction enzymes BamHI and XbaI.

To generate pL0017-MitoKillerRed, we amplified the cDNA of KillerRed by PCR with the forward primer KillerRed-fw 5′-TGGATCCATGGGTTCAGAG-3′ and the reverse primer KillerRed-rev 5′-TTCTAGATTAATCCTCGTCGCTACC-3′ (Table 1) with the KillerRed plasmid from Prof. Markus Meissner as a template. The cDNA of KillerRed was subcloned via blunt ends into pJET1.2 and the sequence was verified by the sequencing service of Microsynth AG in Balgach, Switzerland. The KillerRed cDNA fragment was excised with BamHI and XbaI. The final vector derived from the linearized p^c^mCherry^MITO^^26^. The sequence of mCherry was replaced with the sequence of KillerRed, which resulted in the pL0017-MitoKillerRed plasmid.

### Parasite strains

All mice and cells were infected with parasite strains that have a *P. berghei* ANKA background. PbmCherry, PbGFP, PbExp1-mCherry as well as PbWT parasites, are phenotypically wildtype. PbmCherry express cytosolic mCherry under the control of the *P. berghei* hsp70 regulatory sequences^41^. PbGFP parasites express GFP in their cytosol under the promoter of the elongation factor 1-alpha (eef1α)^42^. PbExp1-mCherry express the PVM marker Exp1 tagged with mCherry under the liver stage specific promoter LISP2^3^.

### Generation and analysis of PbMitoKillerRed

Detached cells (DCs) of PbWT parasites were produced and transfected as described previously^43^. To generate *P. berghei* parasites expressing KillerRed mitochondrially, we generated the plasmid pL0017-MitoKillerRed. The plasmid was linearized with ApaI and SacII before transfecting DCs. The drinking water of infected mice was supplemented with pyrimethamine (Sigma) to select for transfected parasites. To isolate genomic DNA from the parasites, we treated infected red blood cells with 0.05% saponin before using the NucleoSpin Blood QuickPure kit (Macherey-Nagel). Afterwards, we confirmed successful integration of the plasmid into the genome by diagnostic PCRs using GoTaq Flexi DNA polymerase (Promega) (Fig. S2) and primers c-ssu-fw; d-ssu-fw; pL0017-rev, cd-ssu-rev as listed in Table 1.

### Mosquito infection

To infect mosquitoes with *P. berghei* parasites, mice were first intraperitoneally injected with blood stabilates of the desired parasite strain. Parasites were allowed to grow inside the mouse until a parasitemia of 1–7% was reached. Following this, 20–50 µl blood of the infected mouse was transferred by intravenous injection into phenylhydrazine-treated mice (200 µl phenylhydrazine; 6 mg/ml in PBS; 2–3 days before). At three to four days post-infection, mice were checked for gametocytes and if there were mature gametocytes, mice were anesthetized for 35 min to allow feeding of 150 female *Anopheles stephensi* mosquitoes. The age of the mosquitoes at the day of blood feed was between 2–8 days post-hatching. Infected mosquitoes were kept at 20.5 °C with 80% humidity.

### Transient transfection of HeLa cells

Before transient transfections of HeLa cells could be performed, cells were harvested by Accutase treatment. Then, 2 × 10^6^ cells were pelleted by centrifugation at 1000 × g for 2 min at room temperature. Cells were resuspended in Nucleofector V solution (Lonza, VVCA-1003) supplemented with 1–3 μg of plasmid DNA. Using the Nucleofector Electroporation Technology (Lonza), HeLa cells were transfected with the program T-28 of the Nucleofector 2b transfection device according to the manufacturer’s instructions. Cells were then seeded according to the experimental setup.

### Transduction of HeLa cells with lentiviruses

HeLa cells stably expressing LAMP1-GFP were generated using lentivirus transduction. The pRRL.LAMP1-GFP lentiviral expression plasmid is based on pRRLSIN.cPPT.PGK-GFP.WPRE. The LAMP1-GFP ORF was amplified by PCR using the forward primer LAMP1-GFP-fw 5′-ATCGGACCGATGGCGCCCCGCAG-3′ and the reverse primer LAMP1-GFP-rev 5′- ATCGGTCCGTTACTTGTACAGCTCG -3′. The cDNA of LAMP1-GFP cloned into the RsrII restriction site of the pRRLSIN.cPPT.PGK-GFP.WPRE replacing the original GFP sequence.

The VSV-G envelope expressing plasmid pMD2.G (Addgene plasmid #12259), the 2^nd^ generation packaging plasmid psPAX2 (Addgene plasmid #12260) and lentiviral expression plasmid pRRLSIN.cPPT.PGK-GFP.WPRE (Addgene plasmid #12252) were generous gifts from Didier Trono.

Virus production and transduction of HeLa cells was performed as described earlier^7^.

### Indirect immunofluorescence analysis

For IFA analysis, cells were grown on glass cover slips (#0117530, Marienfeld GmbH, Lauda-Königshofen, Germany). After indicated time periods, cells were fixed with 4% paraformaldehyde in phosphate-buffered saline (PBS; 137 mM NaCl, 2.7 mM KCl, 10 mM Na_2_HPO_4_, 1.8 mM KH_2_PO_4_, pH 7.4) for 10 min. at room temperature. Permeabilization was performed for 10 min. in 0.1% triton X-100 (T8787, Sigma-Aldrich, Buchs, Switzerland) plus 0.1% Saponin (Fluka) in PBS. After washing the cells three times with PBS, they were blocked with 10% FCS (in PBS) for 10 min. to cover unspecific binding sites. Afterwards, cells were incubated with primary antibody in 10% FCS (in PBS) plus 0.1% saponin for at least 1 hour. Primary antibodies used were mouse anti-human-LAMP1 (Developmental Hybridoma Bank, clone H4A3; 1:1000); rabbit anti-UIS4 antiserum (provided by P. Sinnis, Baltimore, USA, 1:500); and rabbit anti-TgHSP70 antiserum (provided by D. Soldati-Favre, Geneva, Switzerland, 1:200). Subsequently, cells were incubated with fluorescently labeled secondary antibodies in 10% FCS (in PBS) plus 0.1% saponin for at least 45 min. We used the following secondary antibodies: anti-rabbit Alexa350 (A11069, 1:5000, Invitrogen, Carlsbad, California, USA), anti-mouse Alexa594 (A11032, 1:5000, Invitrogen, Carlsbad, California, USA), anti-rabbit Alexa488 (A11034, 1:5000, Invitrogen, Carlsbad, California, USA). After washing the cells three times with PBS, they were mounted on microscope slides with ProLong^®^ Gold Antifade Mountant (P36930, Thermo Fisher Scientific, Reinach, Switzerland).

### Quantification of LAMP1 association with the PVM

HeLa cells were infected with *P. berghei* parasites and fixed before labeling them with anti-human-LAMP1 and anti-UIS4 antibodies. At least one hundred infected cells were imaged per time point. The images were deconvoluted and the Mander’s colocalization coefficient was evaluated with the Huygens Remote Manager (Scientific Volume Imaging, Hilversum, Netherlands). The graph was drawn with GraphPad Prism version 7.

### Quantification of parasite survival

HeLa cells were seeded in 96-well plates to reach confluency the next day. The next day, they were infected with *P. berghei* parasites. At 2 hpi infected cells were harvested with Accutase treatment and pooled. Afterwards cells were evenly reseeded into six wells for the six time points (24-well plate). Fixation was performed at the indicated time points. At least 460 parasites were counted at 6 hpi in 3 independent experiments. The relative numbers of parasites at the different time points are normalized to the 6 hpi time point.

### Super-resolution microscopy

Stimulated emission depletion (STED) microscopy, was performed using either HeLa cells or primary murine hepatocytes. Cells were seeded on glass cover slips and infected with *P. berghei* sporozoites. At indicated time points, cells were fixed with 4% paraformaldehyde in PBS for 10 min at room temperature. After washing the cells three times with PBS, they were permeabilized for 10 min. (for HeLa cells) or 20 minutes (for primary murine hepatocytes) in 0.1% triton X-100 plus 0.1% saponin in PBS. To cover unspecific binding sites, cells were blocked with 10% FCS (in PBS) for 1 h. Afterwards, cells were incubated with primary antibody in 10% FCS (in PBS) plus 0.1% saponin over night at 4 °C. Primary antibodies used were mouse anti-human-LAMP1 (Developmental Hybridoma Bank, clone H4A3; 1:100); or rat anti-mouse-LAMP1 (Developmental Hybridoma Bank, clone 1D4B; 1:100); and rabbit anti-UIS4 antiserum. On the next day, cells were washed three times with PBS for 5 min. each and incubated with fluorescently labeled secondary antibodies in 10% FCS (in PBS) plus 0.1% saponin for 1 h. Secondary antibodies used were goat anti-rabbit Oregon Green 488 (H + L) (Molecular Probes, Invitrogen, O-11038, 1:250); goat anti-rat Oregon Green 488 IgG (H + L) (Molecular Probes, Invitrogen, O-6382, 1:250); goat anti-mouse Alexa532 IgG (H + L) (Molecular Probes, Invitrogen, A-11002, 1:250); goat anti-rabbit Alexa532 IgG (H + L) (Molecular Probes, Invitrogen, A-11009, 1:250), or goat anti-rabbit Abberior STAR 440SX (Abberior GmbH, 2-0012-003-4, 1:250).

After washing the samples with PBS, they were embedded in Mowiol® 4–88 (Roth, 0713.1) containing 2.5% DABCO® (Roth, 0718.1) antifade.

Stained cells were imaged on a Leica TCS SP8 inverted microscope equipped with the gated STED system and a HC PL APO STED WHITE 100×/1.4 oil immersion objective (Leica Microsystems, Wetzlar, Germany). The 592-nm depletion laser was used, and the images were subsequently deconvolved with the Huygens STED Deconvolution software (Scientific Volume Imaging, Hilversum, Netherlands) and further processed with the image analysis software FIJI.

### Estimation of late endosomes and lysosomes at the PVM

To analyze the functionality of mClover-mRuby-LAMP1 cells were either treated with DMSO or with 10 µM chloroquine for 2 h. To analyze the number of mature lysosomes and LE with mClover3-mRuby3-LAMP1, mClover-mRuby-LAMP1 expressing HeLa cells infected with PbWT (24 hpi) were imaged at the TillPhotonics/FEI iMIC digital spinning disk microscope (FEI Munich) equipped with an APO 60 × 1.35 NA immersion oil Olympus objective and an additional 1.22x magnification lens in front of Andor 897 high speed EMCCD camera. Z stacks were performed with increments of 1 µm. The mClover3 and mRuby3 were sequentially excited for 150 ms with a 488 nm and a 561 nm laser and spectral separated was performed by a 525/50 nm Bright Line single-band bandpass and 594 LP Edge Basic long-pass filter, respectively. Images were deconvolved using the Huygens Remote Manager (Scientific Volume Imaging, Hilversum, Netherlands). The Mander’s colocalization coefficient of green signal and red signal was also assessed with the Huygens Remote Manager (Scientific Volume Imaging, Hilversum, Netherlands).

After performing z stacks, nuclei were stained with Hoechst 33342 (Sigma-Aldrich, B2261) to check if imaged cells were infected.

### Photo-conversion experiments

Photo-conversion experiments were conducted with HeLa cells transiently expressing LAMP1-mEos3.2 infected with PbWT (24 hpi) on the TillPhotonics/FEI iMIC digital spinning disk microscope (FEI Munich) with the widefield modus (WF Stingray F-145B) under stable environmental conditions (37 °C and 5% CO_2_). Time-lapse acquisition was performed with the 60×/1.35 NA oil-immersion objective from Olympus and the Hamamatsu ORCA-R2 camera. The binning was set to 2 × 2 pixel. After acquiring two images, photo-conversion was performed with 15% power of a 405 nm laser diode in a diffraction-limited ROI. The dwell time was set to 1.0 ms/µm^2^ and the line overlap to 75%. Fluorescence was captured every 2 min., excited with 488 nm and 561 nm wavelengths and emission collected through a multi band dichroic 405/490/561/640 nm filter. Intensities were measured with the FIJI Software. Fluorescence intensity was normalized to the first post-activation image.

### Live cell imaging and time-lapse microscopy

Live cell imaging and time-lapse microscopy were performed using a Leica DMI6000B widefield epifluorescence microscope. During imaging, cells were kept at 37 °C in 5% CO_2_. Images were acquired using a Leica HCX PL APO CS 63 × 1.2 water objective and the Leica LAS AF Software, version 2.6.0.7266. Images were processed with FIJI. Imaging was started at 2 hpi and performed for 48 h (from 2–50 hpi).

### GFP quenching through acidification

To analyze if the GFP signal of PbGFP parasites decreases after lysosomal acidification HeLa cells were infected with PbGFP parasites. Cells were treated with 250 nM LysoTracker Red DND-99 (Invitrogen L7528) at 2 hpi. Live cell imaging was started at 3 hpi and performed for 24 h (from 3–27 hpi).

### Induction of ROS production with KillerRed

Activation of KillerRed was conducted on the TillPhotonics/FEI iMIC digital spinning disk microscope (FEI Munich) with the widefield modus (WF Stingray F-145B) under stable environmental conditions (37 °C and 5% CO_2_). Time-lapse acquisition was performed with a 60×/1.35 NA oil-immersion objective from Olympus and the Hamamatsu ORCA-R2 camera. The binning was set to 2 × 2 pixel. Photo-activation was performed with 20% laser power of a 561 nm laser diode in a diffraction-limited ROI after acquiring two pre-activation images. The dwell time was set to 1.0 ms/µm^2^ and the line overlap to 41%. KillerRed was activated at 24 hpi and parasites were imaged overnight (16 h).

### Quantification of LAMP1 rim-associated parasite death

LAMP1-GFP expressing HeLa cells were either infected with PbmCherry or PbMitoKillerRed parasites. Photo-activation was performed at 24 hpi as described and parasites were then imaged overnight (16 h). Control parasites were imaged without photo-activation. Parasites were categorized as LAMP1 rim-associated death, if their size continuously decreased after the appearance of the LAMP1-positive rim. At least 10 parasites were measured for every setting in 3 independent experiments.

### Statistical analysis

Groups were compared by a 2-tailed, unpaired student’s t test. The statistical analyses were conducted using GraphPad Prism version 7 for Mac, GraphPad Software, San Diego, California, USA.

## Supplementary information

Supplementary Figures

Movie 1a

Movie 1b

Movie 1c

## Data Availability

All data generated or analyzed during this study are included in the published article (and its Supplementary Information Files).
