# Supplementary material for: Deciphering host lysosome-mediated elimination of Plasmodium berghei liver stage parasites
Source: Sci Rep. 2019 May 28;9:7967. doi: 10.1038/s41598-019-44449-z (PMC6538699; doi:10.1038/s41598-019-44449-z)
Supplement: Supplementary file 1 — Supplementary Figures [file 41598_2019_44449_MOESM1_ESM.pdf]

Deciphering host lysosome-mediated elimination of *Plasmodium berghei* liver stage parasites

L. Niklaus[1,2], C. Agop-Nersesian[1,3], J. Schmuckli-Maurer[1], R. Wacker[1], V. Grünig[1], V.T. Heussler[1]

[1] Institute of Cell Biology, University of Bern, Bern, Switzerland

[2] Graduate School of Cellular and Biomedical Sciences, University of Bern, Bern, Switzerland

[3] Department of Molecular and Cell Biology, Henry M. Goldman School of Dental Medicine, Boston University, Boston MA, USA

Correspondence to volker.heussler@izb.unibe.ch

Supplementary Figure 1

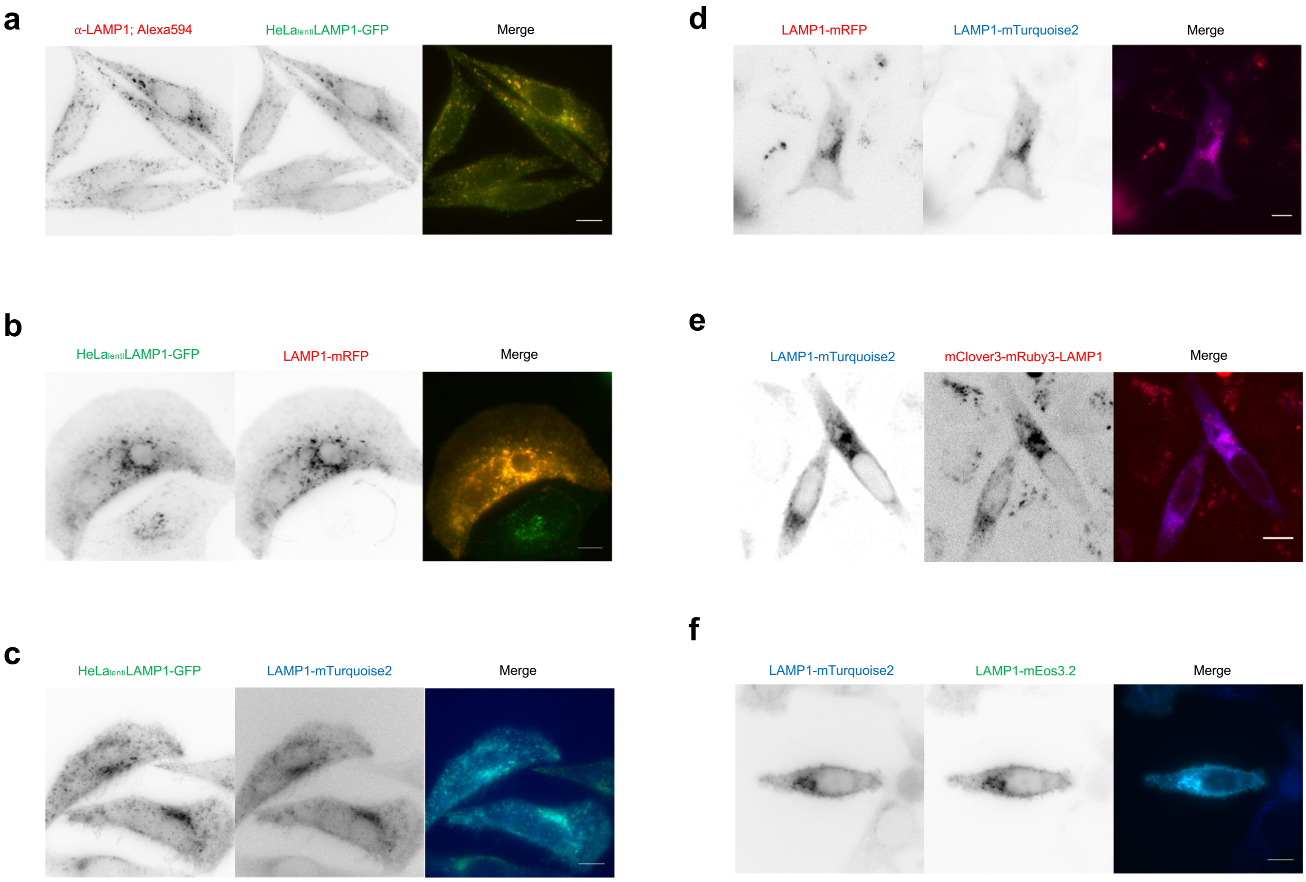

Supplementary figure S1 (a) HeLa cells stably expressing LAMP1-GFP (green) were fixed and antibody stained with α-LAMP1 (red). Colocalization visible in the merged image shows correct localization of LAMP1-GFP. **(b)** HeLa cells stably expressing LAMP1-GFP (green) were transfected with LAMP1-mRFP (red). In the merged image it is shown that the mRFP-tagged LAMP1 localizes correctly. **(c)** HeLa cells stably expressing LAMP1-GFP (green) were transfected with LAMP1-mTurquoise2 (blue). Correct localization of LAMP1-mTurquoise2 can be seen in the merged image. **(d)** HeLa cells were co-transfected with LAMP1-mRFP (red) and LAMP1-mTurquoise2 (blue). The merged image shows that the two tagged versions of LAMP1 colocalize. **(e)** mClover3-mRuby3-LAMP1 (red) was co-transfected with LAMP1-mTurquoise2 (blue). The merged image of transfected HeLa cells shows colocalization. **(f)** HeLa cells were co-transfected with LAMP1-mTurquoise2 (blue) and LAMP1-mEos3.2 (green). The merged image shows that LAMP1-mEos3.2 localizes correctly. Scale bars are 10 μm.

Supplementary Figure 2

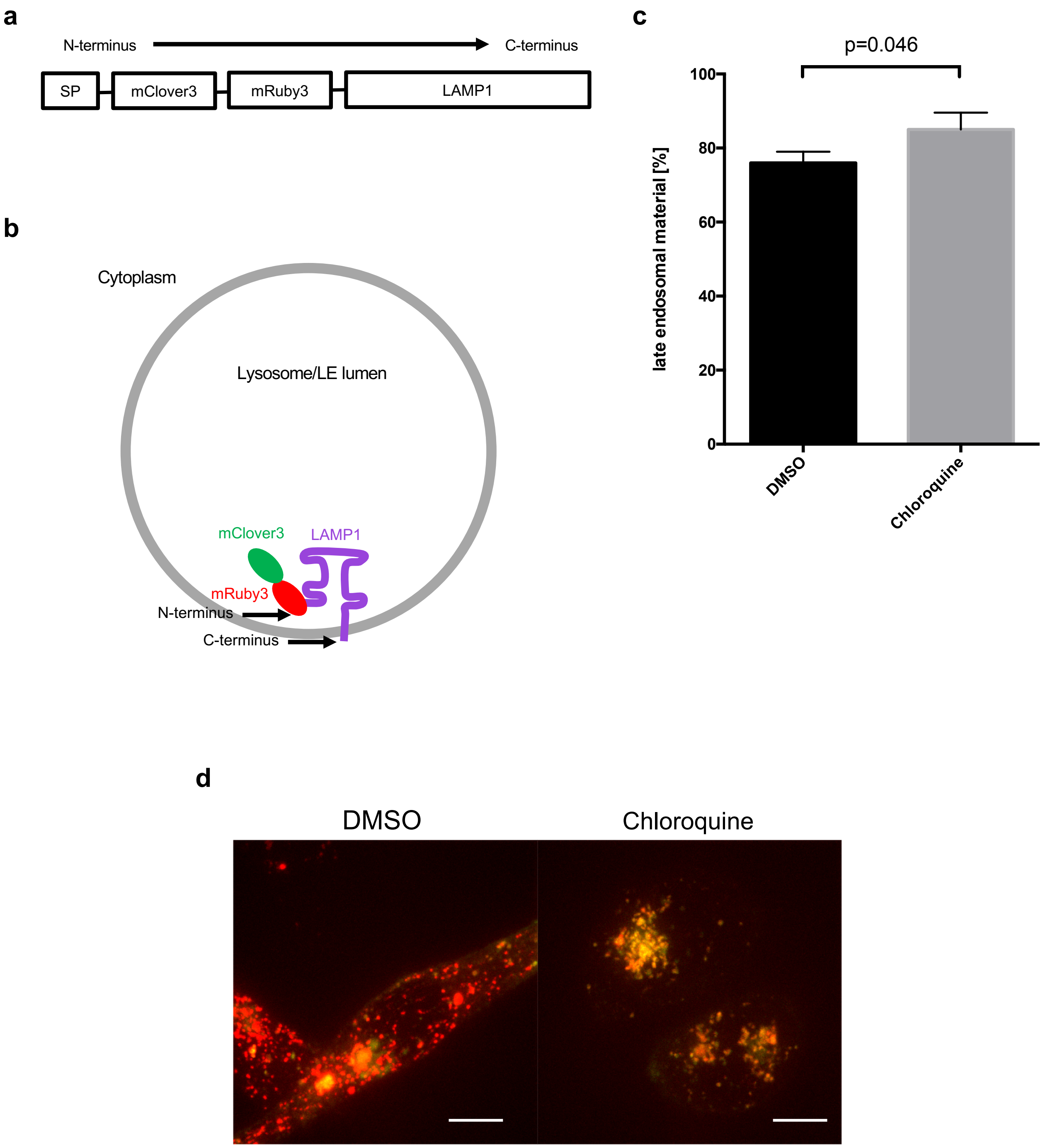

Supplementary figure S2 The pH sensor mClover3-mRuby3-LAMP1. In this tandem construct, two fluorescent proteins with different pH sensitivities were fused to LAMP1. The pKa of the green fluorescent protein mClover3 is at 6.5[40] and therefore its fluorescence decreases in acidic conditions. The red fluorescent protein mRuby3 has a pKa of 4.8[40] and emits fluorescence even in an acidic environment. **(a)** LAMP1 is represented from its N- to the C-terminus. The N-terminus contains a signal peptide (SP). mClover3 and mRuby3 were fused to the N-terminus of LAMP1 right after the signal peptide (SP). **(b)** A lysosome is represented with LAMP1 in its membrane. The N-terminus resides in the lysosomal lumen and the C-terminus points into the cytoplasm. **(c,d)** HeLa cells were transfected with mClover3-mRuby3-LAMP1. One day post-transfection the cells were either treated with DMSO or 10 µM chloroquine for 2h. **(c)** Quantification of the colocalization between green and red signal. Colocalization was evaluated via the Mander's colocalization coefficient. Mander's values are between 0 and 1, where high colocalization equals 1 and therefore means 100% coverage of green signal with red signal. Inhibition of acidification with chloroquine shows that there is stronger green signal compared to cells treated with DMSO. Column graph of three independent experiments with N=10 cells. A student's t test was used to determine the p values. **(d)** Representative images of each treatment are shown. Scale bars are 10 µm.

Supplementary Figure 3

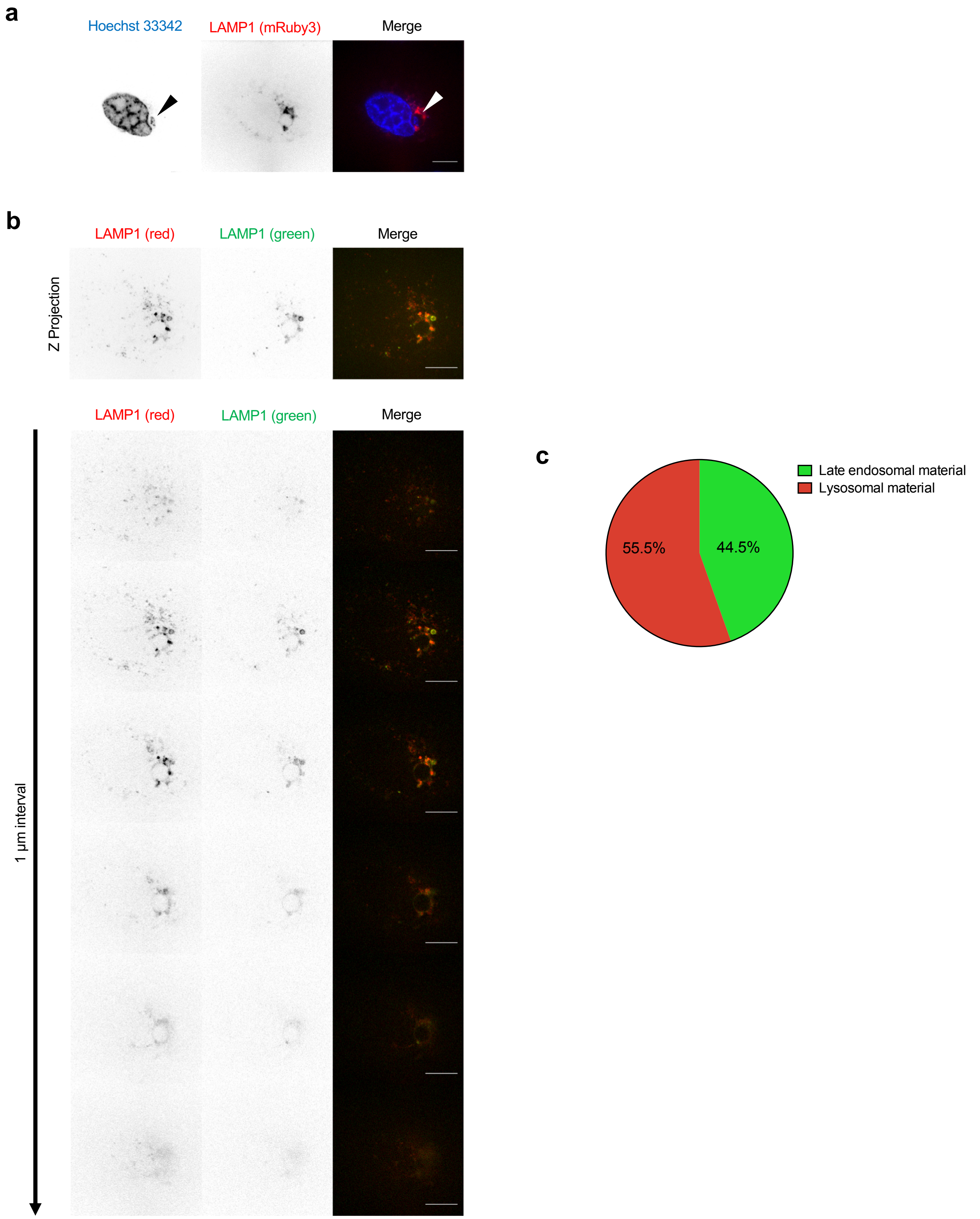

Supplementary figure S3 Parasites associate with mature lysosomes and LE. **(a,b)** HeLa cells were transfected with mClover3-mRuby3-LAMP1 and infected with wild-type *P. berghei* (PbWT). Imaging was performed at 24 hpi. **(a)** Cells were stained with Hoechst 33342 to identify the parasite by its nuclei. **(b)** The same infected cell is represented as in **(a)**. A MAX Intensity Z Projection of a z stack with 1 µm interval was performed to analyze the distribution of late endosomal (red and green) and lysosomal material (red). In the merged image one can recognize that not only late endosomal material (red and green), but also material of mature lysosomes (red) localizes around the parasite. The image of the z stacks are represented below the Z Projection. Scale bars are 10 µm. **(c)** Estimation of late endosomal material (red and green) and lysosomal material (red) around the parasite. Pie chart of late endosomal material and lysosomal material of one representative cell. Mander's colocalization coefficient of green signal compared to red signal of mClover3-mRuby3-LAMP1. Mander's values are between 0 and 1. High colocalization equals 1, while no colocalization equals 0. Colocalization of 1 means 100% coverage of green signal with red signal. Colocalization of green with red signal represents the late endosomal material, while red signal only represents lysosomal material. N = 3 parasites of two independent experiments. Data sets for the other two representative parasites can be found in figure S4.

Supplementary Figure 4

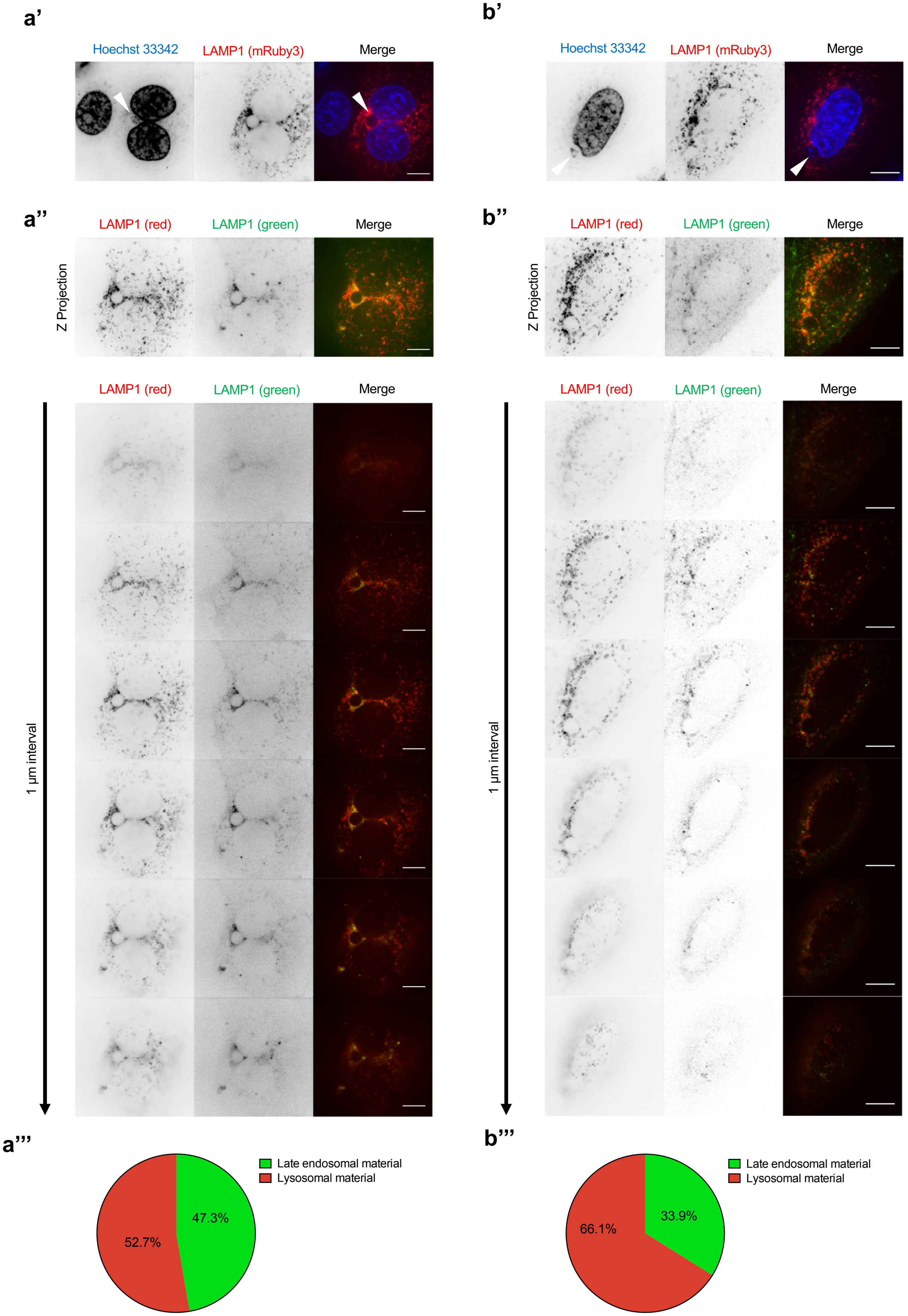

Supplementary figure S4 Mature lysosomes and LE associate around parasites. HeLa cells expressing mClover3-mRuby3-LAMP1 were infected with wild-type *P. berghei* (PbWT) and imaged at 24 hpi. **(a’,b’)** To identify parasites, cells were stained with Hoechst 33342, which allowed to visualize the nuclei. **(a’’,b’’)** The same cells are represented as in **(a’)** and **(b’)**, respectively. Z Projection (MAX Intensity Z Projection) of the z stacks with 1 µm interval were performed to analyze the distribution of late endosomal (red and green) and lysosomal material (red). The merged figures show that not only late endosomal material (red and green), but also material of mature lysosomes (red) associate with the parasites. The figures of the z stacks can be found bellow the Z Projection. Scale bars are 10 µm. **(a’’’,b’’’)** Estimation of late endosomal material and material of mature lysosomes around the parasite. Pie chart of late endosomal material and lysosomal material of the representative cells. Mander’s colocalization coefficient of green signal compared to red signal of mClover3-mRuby3-LAMP1. Mander’s values are between 0 and 1. High colocalization equals 1, while no colocalization equals 0. Colocalization of 1 means 100% coverage of green signal with red signal. Colocalization of green with red signal represents the late endosomal material, while red signal only represents material of mature lysosomes.

Supplementary Figure 5

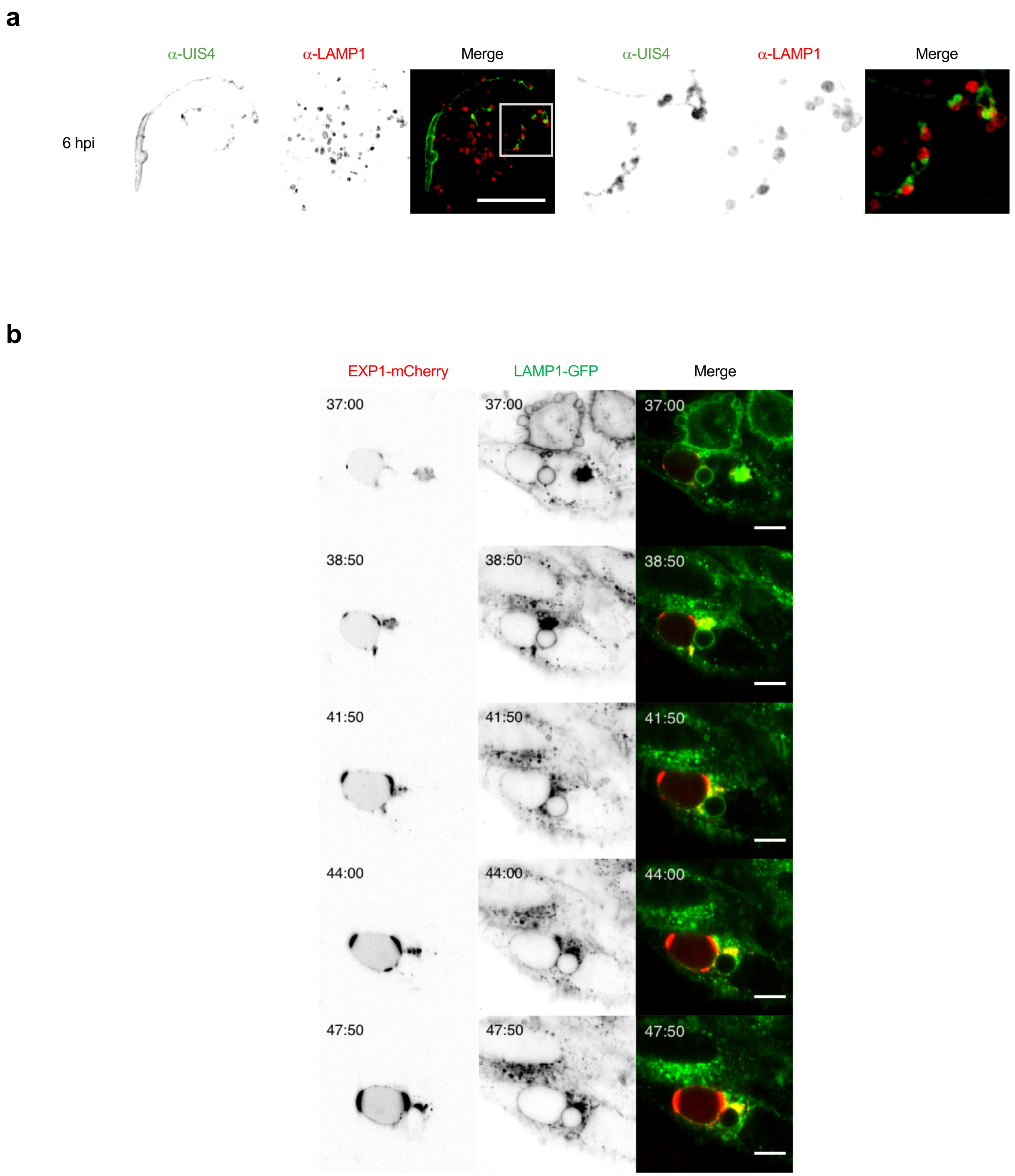

Supplementary figure S5 LAMP1-positive vesicles accumulate at the TVN of early liver stage parasites and late liver schizonts. **(a)** Stimulated emission depletion (STED) microscopy on HeLa cells infected with 6 hpi parasites and stained for the PVM marker UIS4 (Abberior STAR 440SX, represented in green) and the lysosomal marker protein LAMP1 (OregonGreen; represented in red). Enlargement of the indicated boxed part next to the image show vesicles at the TVN. Scale bar is 10 µm. **(b)** Long-term imaging of HeLa cells stably expressing LAMP1-GFP (green) infected with liver schizonts (37 hpi) expressing EXP1 tagged with mCherry under the late liver stage promoter LSP2 (red). Acquisition interval 10 min. Time stamp is hh:mm post-infection. Scale bars are 10 µm.

Supplementary Figure 6

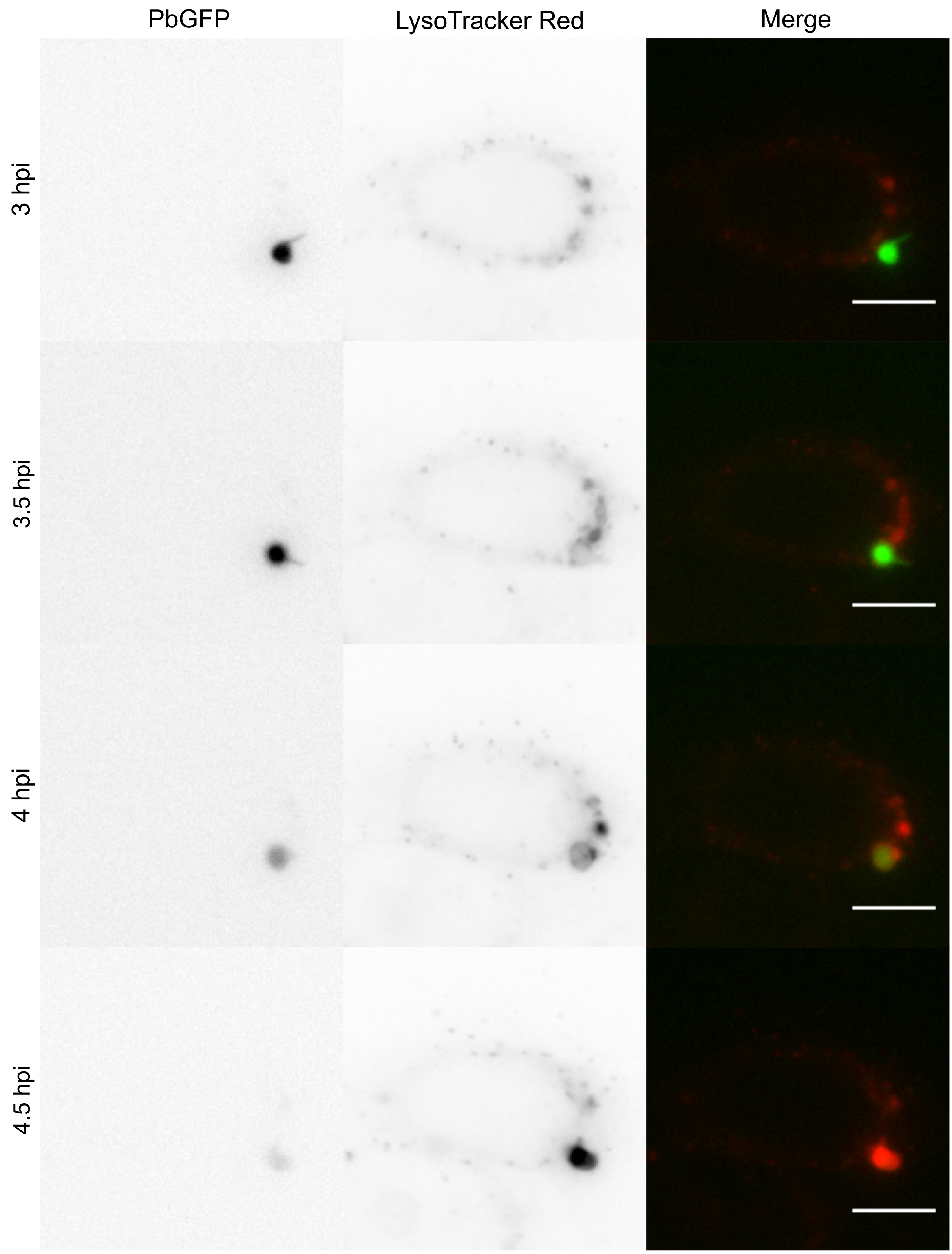

Supplementary figure S6 Quenching of the GFP signal correlates with the appearance of the LysoTracker Red signal. HeLa cells were infected with parasites expressing cytoplasmic GFP (PbGFP, green) and stained with LysoTracker Red (red). As soon as the GFP signal decreases the LysoTracker Red signal intensifies inside the PV of the parasite. Scale bars are 10 µm.

Supplementary Figure 7

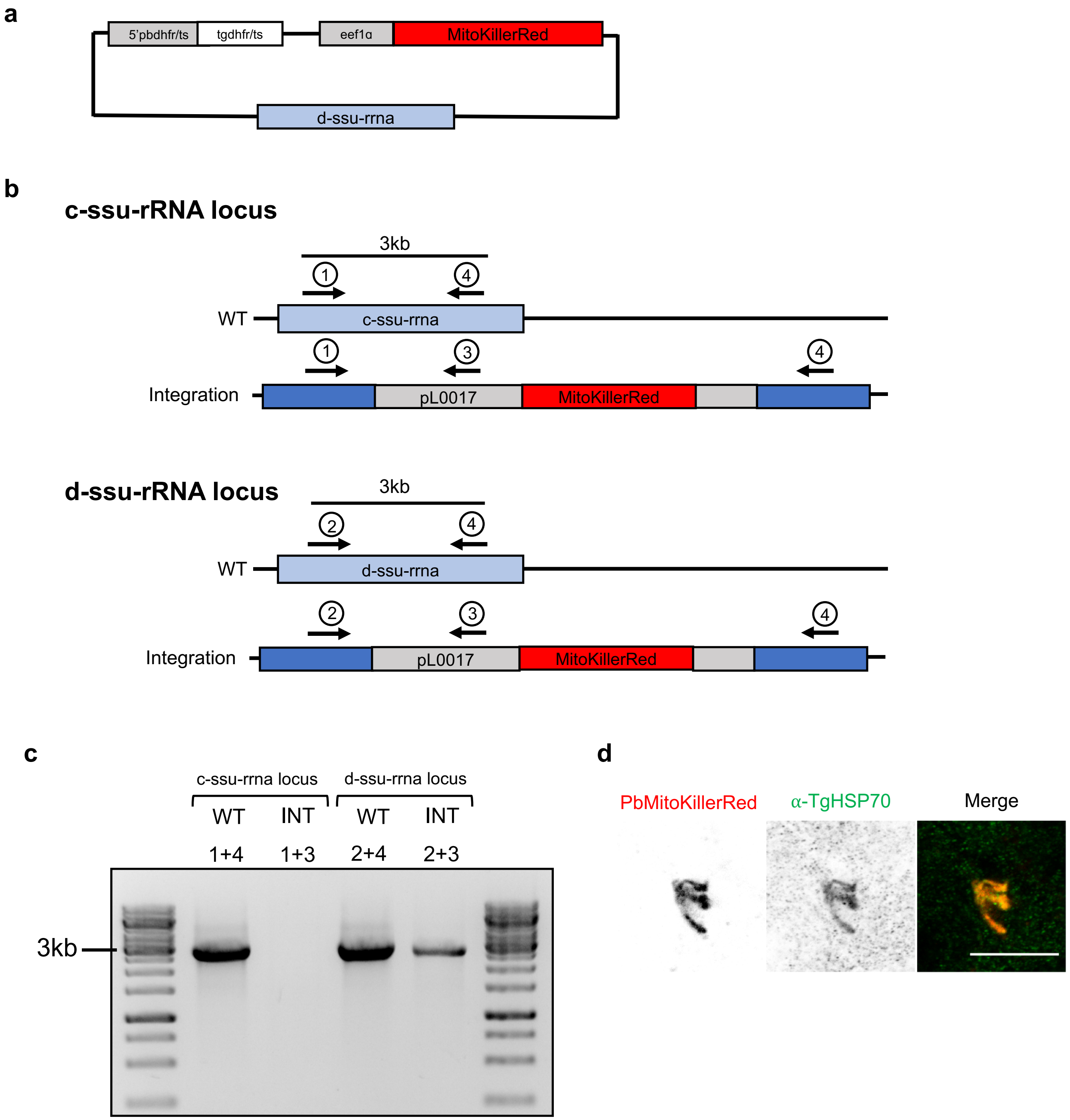

Supplementary figure S7 Integration PCR of pL0017-MitoKillerRed into the d-ssu-rrna locus and mitochondrial localization of KillerRed in parasite. **(a)** Schematic representation of the pL0017-based MitoKillerRed plasmid used to generate PbMitoKillerRed parasites. This vector integrates into the *c*- or the *d-ssu*-rRNA locus via single-crossover recombination and contains a pyrimethamine resistance cassette. **(b)** Diagnostic PCR to prove integration of the vector into the d-ssu-rrna locus. Primer 1 is c-ssu-fw, primer 2 is d-ssu-fw, primer 3 is pL0017-rev and primer 4 is called cd-ssu-rev. Primer sequences are represented in table 1. **(c)** The whole gel is represented and shows the bands of each PCR with the same exposure time. **(d)** HeLa cells were infected with PbMitoKillerRed parasites. Correct mitochondrial localization of KillerRed was proven with α-TgHSP70 (green; Alexa488). Scale bar is 10 µm.

Supplementary Figure 8

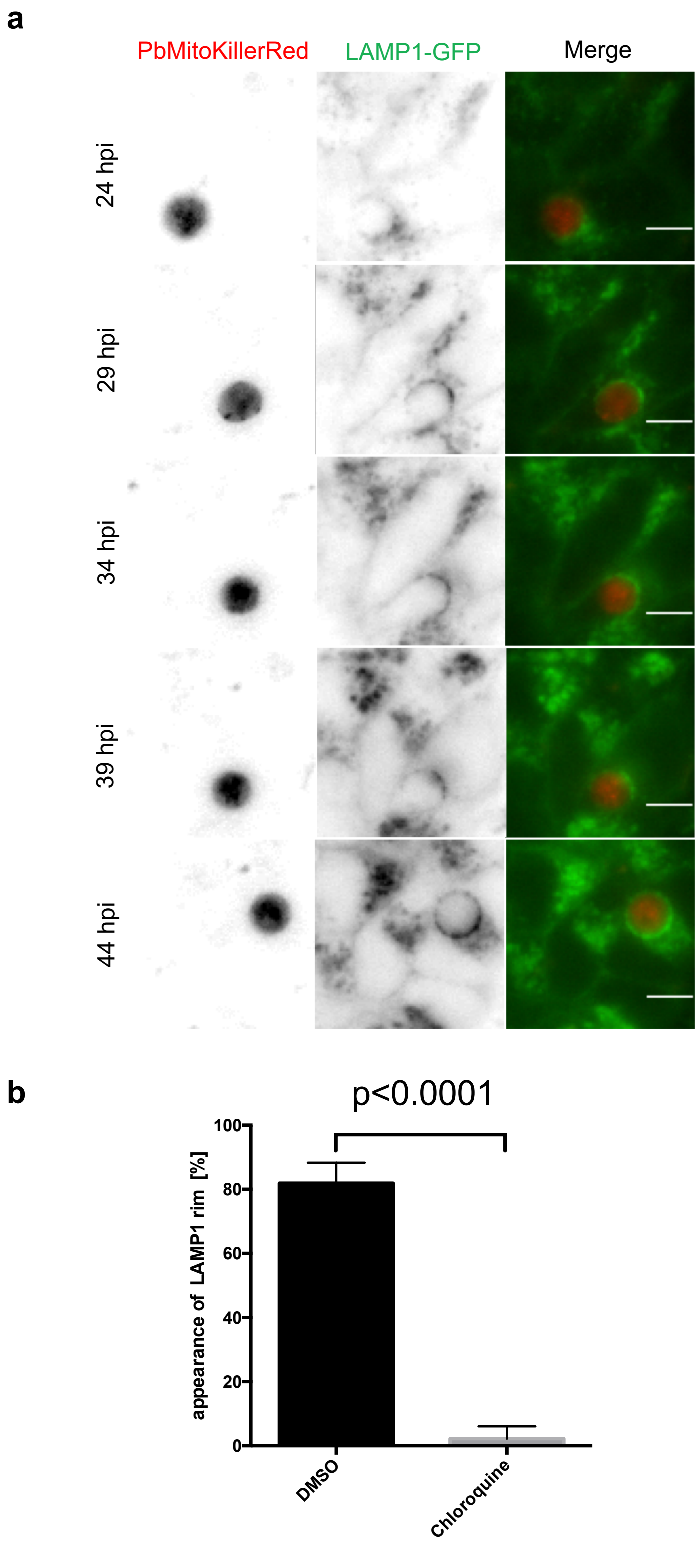

Supplementary figure S8 Lysosomes accumulate in close proximity to the dead parasite after chloroquine treatment but do not fuse. **(a)** PbMitoKillerRed parasites were treated with 10 µM chloroquine starting from 22 hpi and illuminated with green light at 24 hpi. The representative images show that parasites are not eliminated although they do not develop normally. Scale bars are 10 µm. **(b)** Quantification of the appearance of a LAMP1 rim around the parasite. Column graph of relative number of parasites with a LAMP1-positive rim after treatment with DMSO or 10 µM chloroquine and illumination with green light. The graph shows mean and standard deviation of three independent experiments. N ≥ 10 parasites. The p values were determined with a student's t test.

Movie S1 Movies to the Figure 5. Parasite acidification and death correlates with strong rim-like LAMP1 signal around the parasite. **(a)** HeLa cells were transfected with LAMP1-mRFP (red) and infected with GFP-expressing parasites (PbGFP). As soon as LAMP1-mRFP forms a strong signal tightly around the parasite, the acid sensitive GFP disappears. **(b)** HeLa cells stably expressing LAMP1-GFP (green) were infected with mCherry-expressing parasites (PbmCherry). The formation of the strong LAMP1-GFP signal around the parasite takes less than 30 min. This parasite can be seen to be smaller and degraded 8 h later. **(c)** HeLa cells stably expressing LAMP1-GFP (green) were infected with *P. berghei* parasites expressing mitochondrial KillerRed (PbMitoKillerRed, red). LAMP1-GFP signal was intensified tightly around the parasite after killing the parasite. Scale bars are 10 μm.
